# Heterochrony in *orthodenticle* expression is associated with ommatidial size variation between *Drosophila* species

**DOI:** 10.1186/s12915-025-02136-8

**Published:** 2025-02-04

**Authors:** Montserrat Torres-Oliva, Elisa Buchberger, Alexandra D. Buffry, Maike Kittelmann, Genoveva Guerrero, Lauren Sumner-Rooney, Pedro Gaspar, Georg C. Bullinger, Javier Figueras Jimenez, Fernando Casares, Saad Arif, Nico Posnien, Maria D. S. Nunes, Alistair P. McGregor, Isabel Almudi

**Affiliations:** 1 Department of Developmental Biology, University Göttingen, Justus-Von-Liebig-Weg 11, 37077 Göttingen, Germany; 2 Present address: Institute of Clinical Molecular Biology, Christian-Albrechts-University of Kiel, University Hospital Schleswig-Holstein, Kiel, Germany; 3 Department of Biological and Medical Sciences, Oxford Brookes University, Oxford, OX3 0BP UK; 4 Andalusian Centre for Developmental Biology (CABD), CSIC/Universidad Pablo de Olavide/JA. Ctra. de Utrera Km 1, 41013 Seville, Spain; 5 Museum Für Naturkunde, Leibniz-Institut Für Evolutions- Und Biodiversitätsforschung, Invalidenstraße 43, 10115 Berlin, Germany; 6 MRC London Institute of Medical Sciences, London, W12 0NN UK; 7 Centre for Functional Genomics & Department of Biological Sciences, Oxford Brookes University, Oxford, OX3 0BP UK; 8 Department of Biosciences, Durham University, Durham, DH1 3LE UK; 9 Department of Genetics, Microbiology and Statistics, Universitat de Barcelona, Diagonal 643, 08028 Barcelona, Spain; 10 Institut de Recerca de La Biodiversitat (IRBio), Universitat de Barcelona, Diagonal 643, Barcelona, 08028 Spain

**Keywords:** Compound eyes, Ommatidia, *Drosophila*, Evolution, Heterochrony, Development, *Orthodenticle*

## Abstract

**Background:**

The compound eyes of insects exhibit extensive variation in ommatidia number and size, which affects how they see and underlies adaptations in their vision to different environments and lifestyles. However, very little is known about the genetic and developmental bases of differences in eye size. We previously showed that the larger eyes of *Drosophila mauritiana* compared to *D. simulans* are generally caused by differences in ommatidia size rather than number. Furthermore, we identified an X-linked chromosomal region in *D. mauritiana* that results in larger eyes when introgressed into *D. simulans*.

**Results:**

Here, we used a combination of fine-scale mapping and gene expression analysis to further investigate positional candidate genes on the X chromosome. We found earlier expression of *orthodenticle (otd)* during ommatidial maturation in *D. mauritiana* than in *D. simulans*, and we show that this gene is required for the correct organisation and size of ommatidia in *D. melanogaster*. We discovered that the activity of an *otd* eye enhancer is consistent with the difference in the expression of this gene between species, with the *D. mauritiana* enhancer sequence driving earlier expression than that of *D. simulans*. When *otd* expression is driven prematurely during *D. melanogaster* eye development, the ommatidia grow larger, supporting a possible role for the timing of *otd* expression in regulating ommatidial size. We also identified potential direct targets of Otd that are differentially expressed between *D. mauritiana* and *D. simulans* during ommatidial maturation.

**Conclusions:**

Taken together, our results suggest that differential timing of *otd* expression may contribute to natural variation in ommatidia size between *D. mauritiana* and *D. simulans*, which provides new insights into the mechanisms underlying the regulation and evolution of compound eye size in insects.

**Supplementary Information:**

The online version contains supplementary material available at 10.1186/s12915-025-02136-8.

## Background

Understanding the genetic basis of phenotypic diversity is one of the central themes of evolutionary developmental biology. While the causative genes and even mutations underlying evolutionary changes in a growing list of phenotypes have been identified (e. g. [1–10]) and see [11] for a more comprehensive list), we still know relatively little about the genetic basis for the evolution of organ size.

Insects exhibit remarkable variation in the size and shape of their compound eyes, which has allowed these animals to adapt to different environments and lifestyles [12, 13]. This variation greatly affects optical parameters and visual sensation, such as the detection of different intensities, polarisation and wavelengths of light to varying degrees of contrast sensitivity and acuity [13]. Compound eyes vary in the size and/or number of ommatidia: wider ommatidia capture more light, which can increase contrast sensitivity; however, larger interommatidial angles can lead to decreased acuity [13, 14]. Conversely, having many small ommatidia with narrow interommatidial angles can enhance acuity, but this may decrease contrast sensitivity [15–18].

Differences in ommatidia number and size, as well as trade-offs between these structural features of compound eyes, have been described for a range of different insects [19–24]. Furthermore, variation in ommatidia size across the eye within species is also widely documented [21, 25–28]. This size variation suggests areas of regional specialisation, where different visual tasks rely on different parts of the eye.

Several studies have also found extensive variation in eye size within and between closely related species of *Drosophila*, caused by differences in ommatidia number and/or ommatidia size [4, 19, 23, 25, 29–34]. Despite the pervasive variation in eye morphology and the detailed knowledge about eye development in *D. melanogaster* [12, 35–37], little is known about the genetic and developmental bases for variation in eye size even among *Drosophila* species with very few exceptions (e.g. [4, 30]). Interestingly, in these exceptional cases, changes in the timing of the expression of regulatory genes that consequently alter the timing of developmental processes, referred as heterochrony (i.e. differences in the timing or pace of developmental events compared to those in the ancestor [38, 39], have been involved. However, while these reported mechanisms explained eye size differences due to ommatidia number, whether changes in the timing of expression of key transcription factors (TFs) also may play a role in differences in ommatidia size, by modifying the gene regulatory network (GRN) and timing of developmental events controlled by these TF, remained unexplored.

We previously showed that *D. mauritiana* has larger eyes than *D. simulans* mainly due to larger ommatidia [23, 29]. Quantitative trait loci (QTL) mapping of this difference identified a large-effect QTL that explains 33% of the species difference [29]. Introgression of this X-linked region from *D. mauritiana* into *D. simulans* increased ommatidial size and overall eye size of the latter species [29].

Here, we combine high-resolution mapping of this previously characterised X-linked QTL, with transcriptomic analysis of eye-antennal imaginal discs (EADs) of *D. simulans* and *D. mauritiana*, to identify positional candidate genes that are differentially expressed in the developing ommatidia between these two species. We observed a temporal difference in the onset of expression of one of these candidates, the homeobox gene *orthodenticle (otd)*, also known as *ocelliless* (*oc*), and we confirmed that Otd is involved in ommatidia organisation and size determination. We then carried out ATAC-seq to compare putative regulatory regions of *otd* that may underlie the difference in expression of this gene between *D. mauritiana* and *D. simulans.* Our results indicate that differential activity of an orthologous eye enhancer of *otd* between these species results in earlier expression of this homeobox gene during ommatidial maturation in *D. mauritiana*. We hypothesise that this heterochrony in *otd* expression and consequently longer exposure to this transcription factor (TF) in maturing ommatidia in *D. mauritiana* contributes to the development of larger ommatidia in this species.

## Results

### Enlarged ommatidia in *D. mauritiana*

**Fig. 1:**
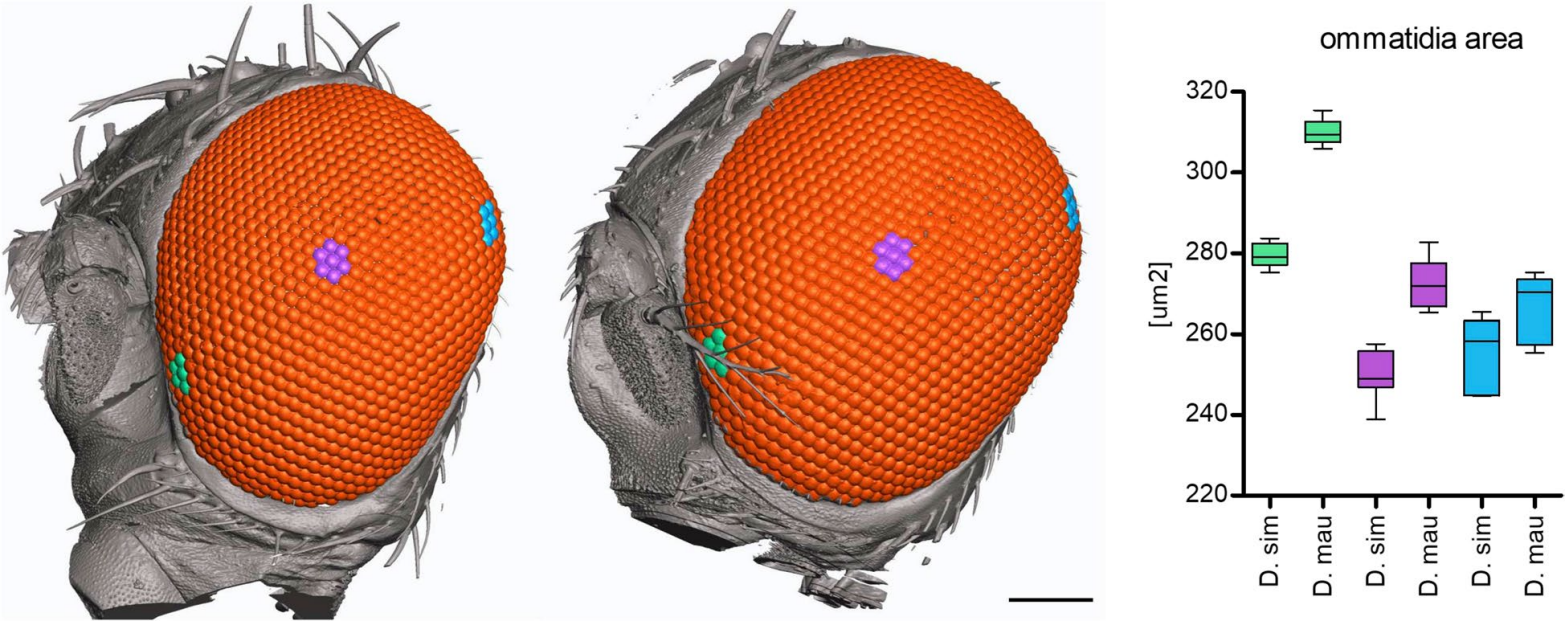
3D reconstruction and ommatidia size measurements from SRμCT data of female *D. simulans* (left) and *D. mauritiana* (right). Facet areas of the ommatidia highlighted in the antero-ventral (green), central (purple) and dorsal-posterior (blue) region of the eye are plotted in corresponding colours (far right). Ommatidia number is 996 for the *D. simulans y*,* v*,* f* and 1018 for the *D. mauritiana* TAM16. Scale bar is 100 μm

We previously found that the central ommatidia of *D. mauritiana* eyes have wider diameters than those of *D. simulans* [23, 29]. To examine whether this phenotypic difference is prevalent in all ommatidia across the eye, we imaged the eyes of a female *D. mauritiana* TAM16 and a female *D. simulans y*,* v*,* f* using synchrotron radiation micro-CT (SRμCT) and measured the facet diameter of ommatidia in different regions of the eye using 3D reconstructions (Fig. 1). We corroborated that while the number of ommatidia is similar between these strains of *D. mauritiana* and *D. simulans* the former has larger facets, as observed for other strains of these two species [19]. This trend is consistent across anterior, central and posterior facets but is particularly pronounced in the antero-ventral region of the eye (Fig. 1 and Additional File 1: Table S1 [23, 29]) again as seen for other strains [19].

### Differentially expressed genes in a candidate region on the X chromosome

**Fig. 2:**
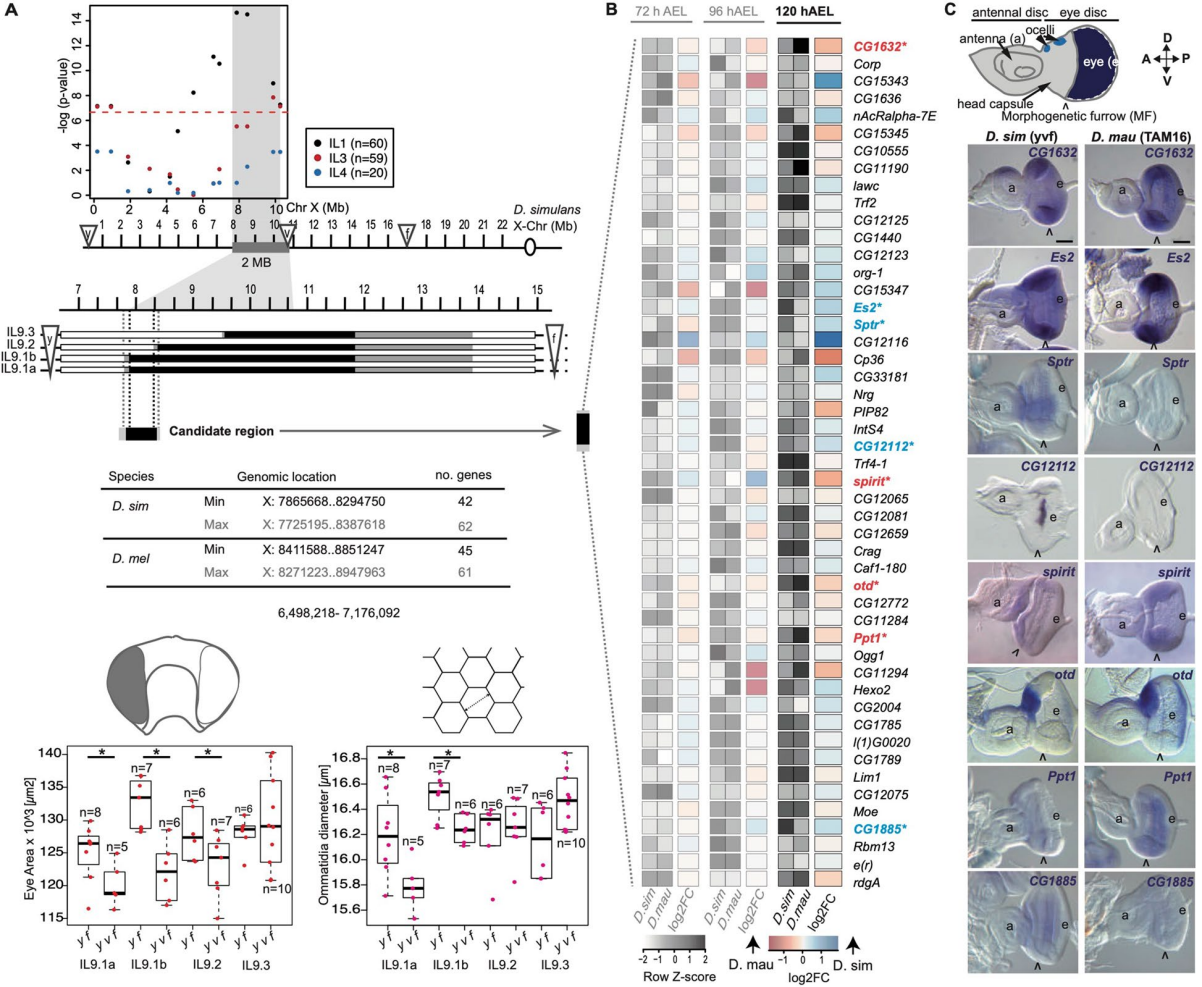
Differential and spatial gene expression. **a** Fine-scale mapping of the X chromosome QTL. Marker-phenotype association in male recombinant progeny (between *y* and *v*) from three replicate introgression lines (IL1, 3, and 4, single-marker ANOVA analysis). Red dashed line indicates the Bonferroni corrected significance threshold of 0.05. Shaded grey area represents a conservative interval of ~ 2 Mb encompassing the X linked QTL. Recombination breakpoints of the new introgression lines (IL9.1–9.3) on the X chromosome (shown for *D. simulans* Flybase R2.02) define the 662 kb candidate region. White, black and grey boxes indicate DNA regions from *D. simulans y*,* v*,* f*, *D. mauritiana* TAM16 or not determined, respectively (the latter define the maximum candidate region). The table indicates the number of protein coding genes that are present in the candidate region in *D. simulans* and *D. melanogaster*. Distribution of eye area (left) and ommatidia diameter (right) measurements by genotype and introgression line. Asterisks indicate levels of significance between genotypes where *p* < 0.05 (Additional File 2: Table S2). **b** Differential expression of 49 protein coding genes located in the introgressed region from **a** and expressed at 72 h AEL, 96 h AEL and 120 h AEL. Genes with significantly higher expression at 120 h AEL in *D. simulans* are highlighted in blue. Genes significantly upregulated in *D. mauritiana* at 120 h AEL are shown in red. **c** Expression of differentially expressed genes at 120 h AEL in L3 EADs of *D. simulans* and *D. mauritiana.* The cartoon illustrates an eye-antenna disc, highlighting the eye territory (in dark blue) where the ommatidia will be determined during late L3 and pupal stages. Open arrowheads indicate the MF, a: antenna, e: eye, A: anterior, P: posterior, D: dorsal, V: ventral, in **c**

Previously we detected a QTL region located between 2.6 Mb and 8 Mb on the X chromosome, which is responsible for 33% of the difference in ommatidia size [29]. Furthermore, introgression of approximately 8.3 Mb of this X-linked region (between the *yellow* (*y*) and the *vermillion* (*v*) loci) from *D. mauritiana* TAM16 into *D. simulans y*,* v*,* f* significantly increased the eye size of the latter, consistent with the direction of the species difference [29]. Further analysis of recombinant males with breakpoints within the introgressed region revealed significant genotype–phenotype associations towards the distal end of the introgressed region near the marker *v*, providing a conservative interval of about 2 Mb wherein the causative loci is likely to reside (Fig. 2a). To map the candidate region to higher resolution we generated introgression lines with breakpoints in the 2 Mb interval and compared eye area and central ommatidia diameter of *y*, *f* male progeny (with some *D. mauritiana* DNA in the 2 Mb interval) to that of their *y*,* v*,* f* sibling males (i.e. without *D. mauritiana* DNA). We found that *y*,* f* males had significantly larger eye size than their *y*,* v*,* f* siblings in introgression lines IL9.1a (one tailed *t* = 2.18, df = 11, *p* = 0.026), IL9.1b (one tailed *t* = 4.54, df = 11, *p* < 0.001) and IL9.2 (one tailed *t* = 2.00, df = 11, *p* = 0.035) but ommatidia diameter was only significantly larger in *y*,* f* males than their *y*,* v*,* f* siblings in introgression lines IL9.1a (one tailed *t* = 2.52, df = 11, *p* = 0.014) and b (one tailed *t* = 3.12, df = 11, *p* = 0.005) (Fig. 2a). Ommatidia number and body size did not differ between *y*, *f* males and their respective *y*,* v*,* f* sibling males for any of the IL lines (Additional File 2: Table S2 and Additional File 3: Fig. S1). These data suggest that the candidate QTL is located in a maximum region of about 662 kb (ChrX: 7,725,195–8,387,618 in *D. simulans*). This mapped region contains 62 protein coding positional candidate genes.

*Drosophila* eyes develop from the eye-antennal imaginal discs (EAD), a monolayered epithelium [40]. Within it, the activity of two morphogens, Hedgehog (Hh) and the BMP2 Decapentaplegic (Dpp), drives a differentiation wave that progresses from the posterior to the anterior region of the primordium and leaves in its wake differentiating retinal cells. These cells then organise into clusters that will form individual ommatidia [35]. The wave-like differentiation implies a temporal axis, in which ommatidial rows are progressively added in the anterior front of the wave. The front of the differentiation wave is characterised by a transient indentation of the epithelium, the so-called morphogenetic furrow (MF). Therefore, the MF is a landmark behind which ommatidial differentiation takes place. To assay which of these candidates are expressed during the generation of ommatidia, we performed RNA-seq experiments on the EADs of 3rd instar larvae (L3). We extracted RNA from *D. mauritiana* and *D. simulans* EADs at three different developmental points: at 72 h after egg laying (AEL; late L2, at the onset of differentiation marked by the MF), when cells in the eye primordium are proliferating and specification of the ommatidial cells has not yet started; at 96 h AEL stage (L3) when the MF has moved about half way across the eye disc and the most posterior ommatidia are already determined, and at 120 h AEL (late L3), when most ommatidia are already determined but their size, structure and shape are not yet finalised [41, 42].

Comparison of the RNA-seq data among these three developmental timepoints showed that transcriptomes of 72 h AEL EADs were the most different in comparison to transcriptomes from both 96 h AEL and 120 h AEL for both species (Additional File 4: Fig. S2 and Additional File 5: Table S3). This reflects the distinctive processes that are occurring at these developmental stages [42]. We next focused on the expression of genes located within the mapped 0.66 Mb X-linked region at 120 h AEL because at this timepoint the posterior ommatidia begin to adopt their final size. Of the 62 genes located in this region, 49 were expressed at least at one of the RNA-seq timepoints and only eight of these genes were differentially expressed between these two species at 120 h AEL (Additional File 5: Table S3): *spirit*, *otd* and *Ppt1* showed higher expression in *D. mauritiana*, whereas *CG1632*, *Es2*, *Sptr*, *CG12112* and *CG1885* were more highly expressed in *D. simulans* (Fig. 2b).

We next performed in situ hybridization experiments of these eight candidate genes to investigate if they are expressed in the eye field where the ommatidia are being assembled. These assays were carried out in both *D. mauritiana* and *D. simulans*, which allowed us to determine whether the differences in expression levels observed in the RNA-seq datasets are related to differences in spatial expression (Fig. 2c). *Sptr*, *CG12112* and *spirit* had no detectable expression in the relevant region posterior to the MF (Fig. 2c). *Ppt1* and *CG1885* were expressed both anterior to and immediately posterior to the MF. *CG1632* and *Es2* were ubiquitously expressed in the eye disc, with no clear regional differences. Finally, as previously shown in *D. melanogaster*, *otd* was expressed in the ocellar region of the EAD and in the most posterior region of the eye field [43]. At 120 h AEL *otd* is already expressed in several rows of the most posterior ommatidia of *D. mauritiana* eye discs, whereas *otd* expression is weakly detected in a smaller posterior region of the eye discs of *D. simulans* (Fig. 2c, Additional File 6: Fig. S3, Additional File 7: Table S4). These results were consistent with our differential expression analysis, as the analyses of spatial expression showed qualitative differences in expression levels for most of the investigated genes. Taken together, these results showed that *otd* is the only differentially expressed positional candidate gene that is expressed in maturing ommatidia (Fig. 2c).

### Differences in *otd* gene expression during eye development between *D. simulans* and *D. mauritiana*

**Fig. 3:**
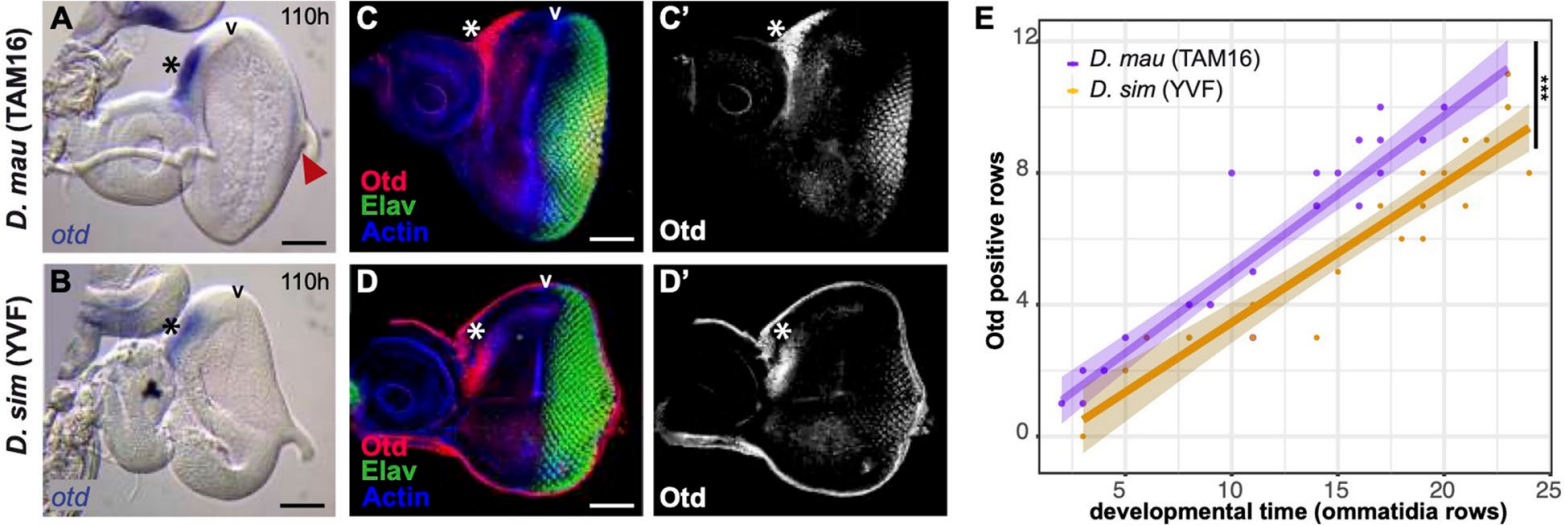
*otd* expression in L3 eye imaginal discs. (a-d) *otd* mRNA at 110 h AEL in *D. mauritiana* (**a**) and *D. simulans* (**b**). Black arrowheads indicate the MF. Asterisks indicate expression in the ocellar region. *D. mauritiana* already has detectable *otd* mRNA at 110 h (red arrowhead). **c**–**d’** Immunostaining showing Otd protein (magenta, **c’**, **d’**) in mature ommatidia (marked in green by Elav) and the ocellar region (asterisk) in *D. mauritiana* (**c**–**c’**) and *D. simulans* (**d**–**d’**). Staining against actin (blue) was used to mark the MF. **e** Plot showing the number of Otd-positive ommatidia rows (*y*-axis) at different developmental time points (*x*-axis, developmental points inferred by number of rows of ommatidia). Asterisks represent statistical significance *p* < 0.001 of the Analysis of Covariance (ANCOVA) (*F *_*1*,*47*_ = 30.3, *p*-value = 1.48 × 10^−06^). Scale bars: 50 μm. Anterior regions of the EADs are on the left and posterior on the right

Our results suggested that *otd* transcription in the maturing ommatidia initiates earlier in *D. mauritiana* than in *D. simulans* (Fig. 2c). To investigate this further, we performed additional in situ hybridizations at 110 h AEL to compare the onset of *otd* expression in the developing ommatidia of these two species. At this developmental stage, we found that *otd* is already transcribed in *D. mauritiana* eye discs, whereas there was no detectable expression in *D. simulans* discs (Fig. 3a, b, Additional File 8: Fig. S4). To confirm this heterochrony in *otd* expression, we performed immunostainings against Otd protein in developing eye discs (Fig. 3c, d). As an exact staging based on hours AEL may not recapitulate subtle interspecific differences in developmental timing, we counted the number of ommatidial rows that were already specified (i.e. with positive Elav staining) as a proxy of developmental stage and then which of these ommatidial rows showed Otd expression. We observed that *D. mauritiana* eye discs displayed more Otd-positive ommatidia than *D. simulans* eye discs at the same stage (Elav positive ommatidia rows, Fig. 3e, Additional File 9: Table S5, *F *_*1*,*47*_ = 30.3, *p*-value = 1.48 × 10^−06^). Thus, cells in maturing ommatidia are exposed to the activity of Otd for longer in *D. mauritiana* since the expression of this protein extends into the pupal stage of both species (Additional File 10: Fig. S5).

### Otd is required for the correct arrangement and size of ommatidia in *Drosophila*

It was previously shown in *D. melanogaster* that *otd* is expressed in photoreceptor cells and required during pupal stages for morphogenesis of their rhabdomeres, and subsequently rhodopsin expression, as well as for the synaptic-column and layer targeting of the photoreceptors [43–45]. We carried out further analysis of *otd* function during eye development using RNAi knockdown and by generating mitotic clones of homozygous *otd* mutant cells. Decreasing *otd* mRNA by overexpressing an *otd-miRNA* construct in cells posterior to the MF [46] resulted in defects in the final ommatidial organisation. These defects were rescued by adding a copy of *UAS-otd* (Additional File 11: Fig. S6a-c), supporting the specificity of the RNAi-mediated *otd* attenuation. Loss of *otd* in clones resulted in disorganised ommatidia with perturbed shapes and sizes—often smaller than the ommatidia of controls (Additional File 11: Fig. S6d).

To test whether an acceleration of the onset of *otd* expression could cause an increase in ommatidial size, we drove a UAS-*otd* transgene with the *ato3’FL-GAL4* line, which drives the expression of *otd* in the MF and early photoreceptors [47], and therefore earlier than the onset of endogenous *otd* expression. Using one-way ANOVA, we observed that there are significative differences between the experimental genotype (*“ato* > *otd”*) and the two controls (“*otd;* + *”*, and the parental line “*ato* > + *”*; *F*_*7*,*95*_* p*-value = 0.0015)*.* We also performed a Tukey's HSD to identify the differences between the three groups and concluded that the ommatidium area was significatively larger than each of the controls (*p* = 0.0154 and *p* = 0.0052, respectively) when we overexpressed *otd*, while the area of controls did not differ to each other (*p* = 0.9004) (Additional File 11: Fig. S6, Additional File 12: Table S6). Overall, these results show that *otd* expression in the photoreceptor cells of maturing ommatidia is required for the proper regulation of ommatidial organisation and size, with earlier expression resulting in larger ommatidia.

### Differences in chromatin accessibility in the *otd* locus during eye development between *D. simulans* and *D. mauritiana*

**Fig. 4:**
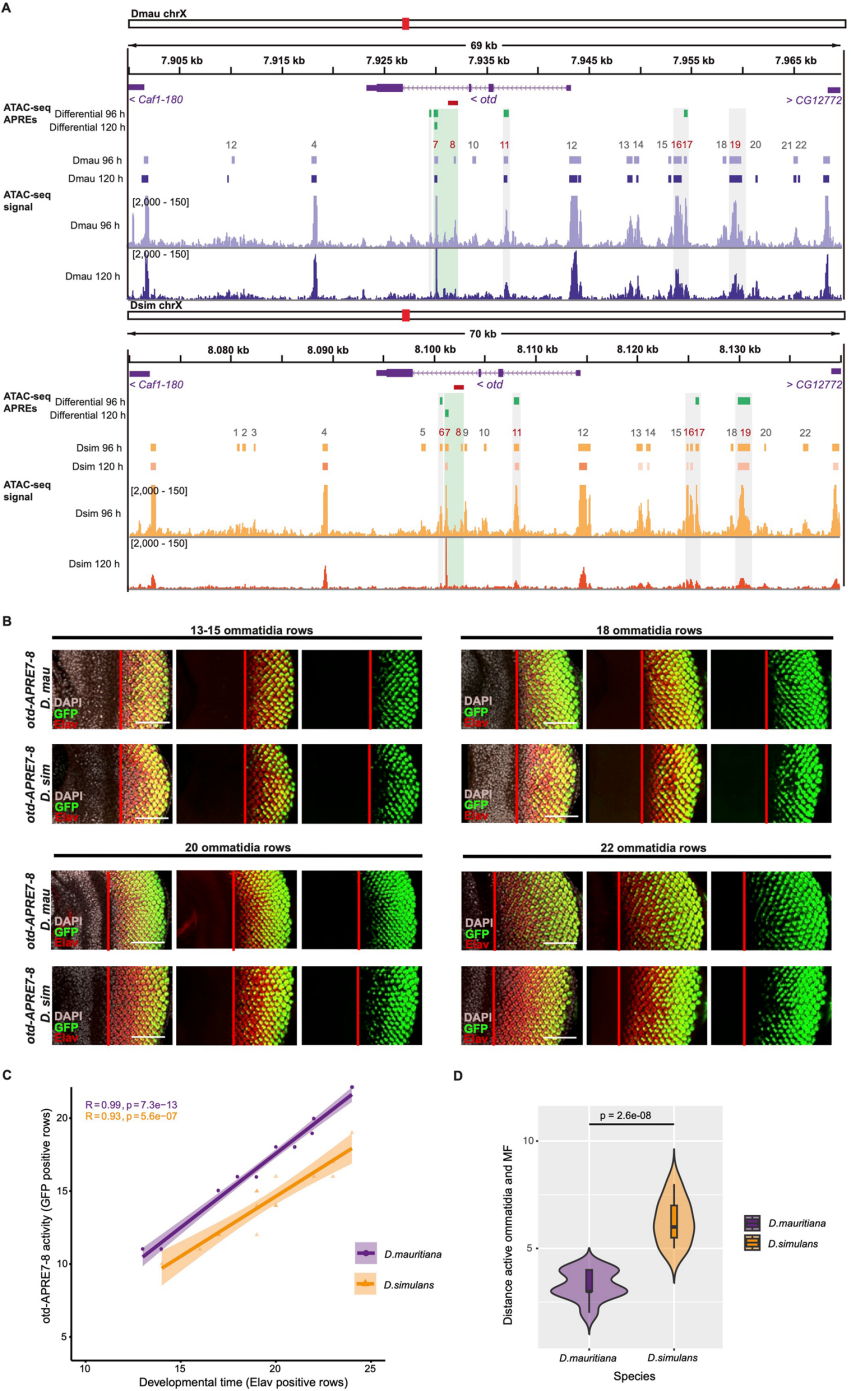
Chromatin accessibility at the *otd* locus. **a** Chromatin accessibility profiles at the *otd* locus at 96 h AEL and 120 h AEL in *D. mauritiana* and *D. simulans* EADs*.* Purple and orange boxes indicate APREs detected in *D. mauritiana* and *D. simulans*, respectively, when mapped against their own reference genome. Green boxes highlight APREs with differential accessibility, identified by re-mapping and peak calling of *D. mauritiana* datasets against *D. simulans* reference genome and *D. simulans* datasets against *D. mauritiana* reference genome, using the "liftOver" tool and MACS2. Red boxes represent the region corresponding to the *D. melanogaster otd*^*uvi*^ allele region. **b** *D. simulans* and *D. mauritiana otd-APRE7-8* enhancer activity in 3rd instar larvae eye imaginal discs. Red bar: limit between MF and differentiated photoreceptors/eye. Scale bars: 50um. Grey: DAPI, red: Elav, green: GFP. **c** Plot showing rows with full enhancer activity (*y*-axis) activated by *otd-APRE7-8* at different developmental time points (*x*-axis, developmental points inferred by number of ommatidia rows) for both species. **d** Violin plots showing the distance between the MF and the rows with full *otd-APRE7-8* activity (first row counting from the posterior margin of the disc with more than 90% of the ommatidia positive for GFP signal). The *D. mauritiana otd-APRE7-8* is active earlier during the differentiation of ommatidia

Our mapping and expression analyses indicate that the differences in *otd* expression likely contribute to differences in ommatidia size between *D. simulans* and *D. mauritiana.* Given that there is no amino acid difference in the Otd homeodomain between our focal strains of *D. simulans* and *D. mauritiana* (Additional File 13: Fig. S7), our data suggest that the causative changes reside in *otd* regulatory regions. Due to the microsyntenic conservation between *D. melanogaster*, *D. simulans* and *D. mauritiana*, we considered the regulatory landscape of *otd* as the region between its two flanking genes, *Caf1-180* and *CG12772*, revealed by the presence of a topological associated domain (TAD) in the corresponding *D. melanogaster* region (a region of 69 kb in *D. mauritiana* and 70 kb *D. simulans*, Fig. 4a, Additional File 14: Fig. S8).

To investigate the regulation of *otd* in the developing eyes of *D. simulans* and *D. mauritiana* further, we performed ATAC-seq [30, 48] on *D. simulans* and *D. mauritiana* EADs at 96 and 120 h AEL. We mapped our datasets against their reference genomes and also against the other species genome (see the “Methods” section) to detect common, differentially accessible and species-specific regulatory regions (Fig. 4a). The ATAC-seq peak calling of the four datasets (two developmental stages and two species) revealed a total of 22 APREs (Associated Putative Regulatory Regions) in the *otd* locus, all of which were located within alignable orthologous regions in the two species (Fig. 4a).

Five of these peaks showed significant differences in accessibility between *D. mauritiana* and *D. simulans* at 96 hAEL and/or 120 hAEL: APRE 6 (*D. sim* chrX*:* 8,100,587- 8,100,808, padj = 0.00155; *D. mau* chrX*:* 7,929,372–7,929,595, padj = 0.000877), APRE 7 (*D. mau* chrX*:* 7,929,910–7,930,230, padj = 1,85E-06) and APRE 11 (*D. sim* chrX*:* 8,107,881–8,108,402, padj = 0.00976; *D. mau* chrX*:* 7,936,681–7,937,179, padj = 0.0143) in the 3rd and 1st introns of *otd*, respectively, and APREs 17 (*D. sim* chrX*:* 8,125,765—8,126,100, padj = 0.0418; *D. mau* chrX*:* 7,954,305–7,954,661, padj = 0.0331) and 19 (*D. sim* chrX*:* 8,129,876—8,131,170, padj = 0.0317) located upstream of *otd* (Fig. 4a, Additional File 15: Table S7).

To test the activity of these APREs in L3 EADs, we generated *D. melanogaster* lines containing the APRE 6, 7–8, 11, 16–17, and 19 sequences from *D. mauritiana* followed by GAL4 and crossed them to UAS-GFP (see the “Methods” section, Fig. 4b and Additional File 16: Fig. S9). APREs 6 and 11 showed no activity in the EADs, which we confirmed using reporters with the equivalent *D. simulans* sequences (Additional File 16: Fig. S9). APREs 16–17 and 19 drove GFP expression anterior to the MF, in the ocellar domain and some regions of the antennal disc (Additional File 16: Fig. S9, Additional File 17: Table S8). Finally, APRE7-8 drove expression in the posterior of the eye disc, in maturing ommatidia, in a similar pattern to endogenous *otd* expression, and consistent with APRE 7 and APRE 8 demarcating the 2.5 kb region corresponding to the *D. melanogaster otd*^*uvi*^ allele previously identified as an *otd* eye enhancer using reporter assays [43] (Fig. 4a and Additional File 18: Fig. S10). We therefore compared the activity of the *D. mauritiana* and *D. simulans* APRE 7–8 sequences, and we observed differences in the onset of activity of these reporter lines. According to mRNA and protein Otd expression, *APRE7-8-GAL4* from *D. mauritiana* activated GFP expression earlier than the reporter with the *D. simulans* sequence (Fig. 4b–d) consistent with the difference in endogenous *otd* expression between these species. These results suggest that differences in the *D. mauritiana* and *D. simulans* APRE 7–8 sequences underlie the difference in *otd* eye expression between these two species. Note that we also tested the activity of the *D. melanogaster* APRE 7–8 sequence and observed it was similar to the *D. simulans* sequence rather than that of *D. mauritiana* consistent with the derived larger ommatidia of the latter species (Additional File 18: Fig. S10).

We aligned the orthologous sequences of the 2.5 kb APRE 7–8 region from different *D. mauritiana*, *D. simulans* and *D. melanogaster* strains and found seventeen potentially fixed differences (thirteen SNPs and four short indels) in *D. mauritiana* (Additional File 19: Dataset S1, Additional File 20: Fig. S11). Interestingly, most of these mutations lie in a 1.5 kb sub-fragment of this enhancer identified by Vandendries et al. (1996) and several fall in predicted binding sites for Sine oculis, Cut and Otd itself (as well as other transcription factors) (Additional File 20: Fig. S11).

### Differences in Otd targets during eye development between *D. simulans* and *D. mauritiana*

**Fig. 5:**
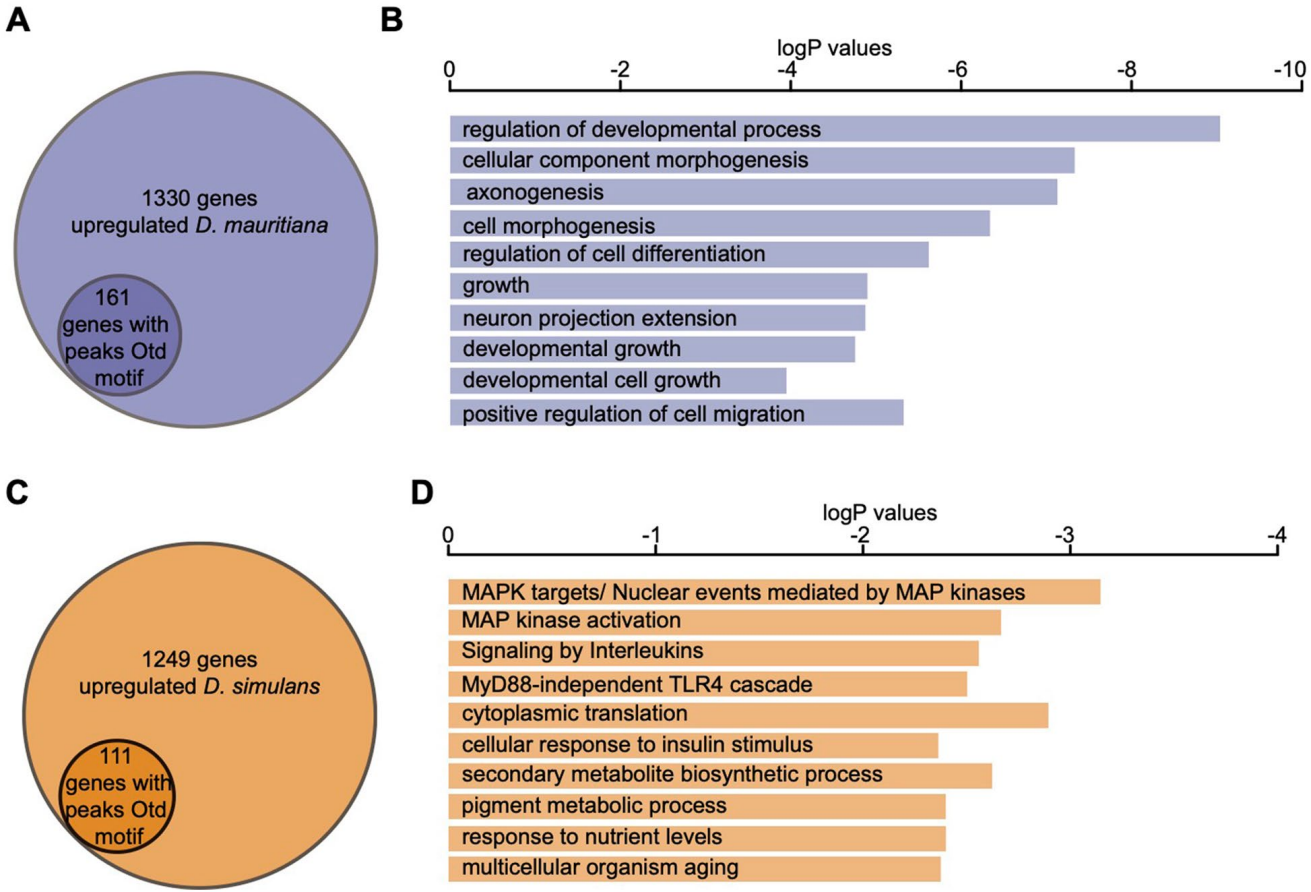
Otd downstream targets. **a** 161 genes upregulated in *D. mauritiana* have an associated peak that contains at least one Otd motif. **b** GO enrichment for those genes with Otd motifs in *D. mauritiana* (**c**) 111 genes upregulated in *D. simulans* have an associated peak that contains Otd motif. **d** GO enrichment for those genes with Otd motifs in *D. simulans*

Next, we investigated whether differences in the onset of expression of *otd* between *D. simulans* and *D. mauritiana* promoted further changes in the expression of downstream genes. To this end, we searched for the Otd-binding motif in accessible chromatin regions of genes expressed during eye development. Based on this analysis, we found 1148 putative Otd target genes. We next examined which of these accessible chromatin regions were associated with genes that were differentially expressed in our transcriptome datasets. We found that 161 APREs of the 1330 genes that are upregulated in *D. mauritiana* contained Otd binding motifs, and 111 out of 1249 genes upregulated in *D. simulans* had associated peaks containing Otd binding motifs (Fig. 5a, c, Additional File 21: Table S9).

We then performed Gene Ontology (GO) term enrichment analysis for these differentially expressed genes with accessible chromatin containing Otd binding motifs. The *D. mauritiana* dataset exhibited enrichment in terms related to developmental processes, cell morphogenesis, axonogenesis, regulation of differentiation or growth, among others (Fig. 5b, Additional File 21: Table S9). By contrast, genes that were upregulated in *D. simulans* with associated Otd-peaks were enriched in terms such as the MAP kinase network, signalling by interleukins and cellular response to insulin stimulus (Fig. 5d, Additional File 21: Table S9).

## Discussion

While much is known about the specification and differentiation of ommatidia, very little is known about the regulation and evolution of their size (although see [49]). To investigate the genetic basis of the difference in ommatidia size between *D. mauritiana* and *D. simulans*, we carried out high-resolution introgression mapping of a previously identified X-linked QTL that explains about 33% of the difference in eye size between these two species [29]. In this region, we identified eight positional candidate genes whose expression in the developing EADs differed between *D. mauritiana* and *D. simulans*. Our analysis of the spatial expression of these eight genes showed expression in the region of the MF (*CG12112*), as well as genes with broad expression in the presumptive retinal region (*CG1632*, *Es2*), which could contribute to eye size differences between species. However, we decided to focus on factors expressed in maturing photoreceptors in the posterior portion of the disc. Based on these criteria *otd* was the best candidate gene in this region for contributing to the difference in ommatidia size and thus overall eye size between these species.

*otd/Otx* genes play several important roles during eye development in both invertebrates and vertebrates [43, 50–53]. During *Drosophila* eye development, Otd regulates genes for cell adhesion and cytoskeletal organisation, which is essential for the correct development of the photoreceptor cells and ommatidia maturation as well as subtype specification through regulation of rhodopsin expression [44, 51, 53, 54]. Mutations in *otd* perturb morphogenesis of the photoreceptor cells [43, 44]. Intriguingly, the removal of photoreceptor cells changes ommatidia size [55]. We suggest that although *otd* is not expressed in the lens-secreting cone cells, it indirectly affects the organisation of these cells and thus ommatidia size through regulating the maturation and organisation of the underlying photoreceptor cells. We have shown that knockdown or loss of *otd* in *D. melanogaster* perturbs ommatidia size specification, but it remains to be directly tested if variation in the expression of this gene underlies larger and smaller ommatidia in *D. mauritiana* and *D. simulans* respectively and if *otd* contributes to the observed variation in ommatidia size in different regions of *Drosophila* eyes [19, 25]. However, the fact that early onset of *otd* expression in *D. melanogaster* using the GAL4/UAS system results in larger ommatidia strongly supports this hypothesis.

Changes in developmental timing, or heterochrony, have played an essential role in the evolution of morphologies in multiple taxa [38, 56, 57]. Classically, the term heterochrony has been used to refer to differences in the timing of developmental events and several examples of heterochrony have been described [58–61]. Most of these characterised cases showed that the genetic basis lies upstream of the mechanism responsible for the heterochrony, such as changes in proliferating rates, differences in the initial size of the primordium or distinct rates of protein stability and biochemistry [62–65]. Heterochronic shifts can also occur as a direct consequence of the causative genetic change, such as those that affect regulatory regions altering the timing of gene expression [4]. Although differences in gene expression of single TFs have the potential to completely modify the subsequent GRN, the relative contribution of such direct heterochrony in generating morphological diversity remains unknown. Our data indicate that *otd* is actually expressed earlier during ommatidial maturation in *D. mauritiana* compared to *D. simulans*, and that premature expression of *otd* results in larger ommatidia, when assayed in *D. melanogaster.* This suggests that cis-regulatory changes in *otd* lead to ommatidial cells being exposed to Otd for longer or earlier in *D. mauritiana.* Globally considered, our results support the notion that temporal variations in the onset of *otd* expression, deriving from the heterochronic action of species-specific enhancers, may contribute to the evolution of ommatidial size. The particular mechanism by which earlier (or longer) expression of *otd* leads to larger lenses (and presumably also larger ommatidia in general) is not well understood, although our results suggest that some rewiring of the Otd-controlled GRN may have happened in the intervening evolutionary period since the common ancestor of *D. mauritiana* and *D. simulans* diverged. Therefore, the evolutionary difference that we attribute to a heterochronic shift in *otd* expression could be coupled to changes in the wider GRN. Together with Ramaekers and colleagues (2019), our study shows how morphological diversity in closely related species may be achieved by subtly altering the temporal expression of a single TF. Importantly, in both cases, these transcription factors, Ey and Otd, act upstream in the GRN controlling the developmental process, thus changes in their expression may promote major differences in downstream effectors.

Further exploration and comparison of the regulatory landscape of *otd* between *D. mauritiana* than *D. simulans* allowed us to identify an intronic region in the *otd* locus, APRE 7–8, corresponding to the orthologous sequence of the previously known “eye enhancer” from *D. melanogaster* [43, 66, 67]. The *D. mauritiana* sequence reporter line showed earlier activity than *D. simulans* sequence, suggesting that this region is responsible for the differences in the timing of *otd* expression and, therefore, function. Further work is needed to identify the TFs that directly bind to this enhancer and the changes in *D. mauritiana* sequence that underlie the differential activity of this element in this species (Additional File 16: Fig. S9).

We also investigated how these changes in *otd* expression might alter target gene expression to change ommatidia size. We identified a set of genes that are differentially expressed between these two species when the ommatidia are acquiring their final size that may be acting downstream of Otd, as they have accessible chromatin regions containing putative Otd binding motifs. We compared this set of genes to known and putative targets of Otd which have been characterised later in eye development during pupal stages [44, 51]. This comparison showed that a subset of genes with altered expression in *otd* mutants are also differentially expressed between *D. mauritiana* and *D. simulans* in late L3. Among other TFs (*Dve*, *vnd*, *MED10*) we identified several genes involved in phototransduction (e.g. *slo*, *Slob*, *ninaG*, *inaD*, *ninaA*) and genes encoding cytoskeleton and adhesion proteins (*Act88F*) (Additional File 21: Table S9). In addition, we found several genes involved in regulation of growth (e.g. *DAAM*, *Thor*, *Frizzled 2*, *kibra* or *Ankyrin 2*) which could have an impact in ommatidia size. This further suggests that the network downstream of Otd varies between these two species and that ultimately, these changes in the GRN, promoted by an early expression of *otd* in *D. mauritiana*, result in differences in ommatidia size between *D. mauritiana* and *D. simulans*.

Interestingly, we recently showed that *D. mauritiana* has higher contrast sensitivity than *D. simulans* [19], while the latter species has greater spatial acuity consistent with the differences in ommatidia size between these species. The trade-off between contrast sensitivity and acuity is heavily influenced by various aspects of visual ecology, such as habitat type, circadian activity patterns and lifestyle. Thus, substantial functional consequences with strong ecological implications could be linked to changes in the expression of individual genes such as *otd*.

## Conclusions

Our data suggest that changes in the timing of *otd* expression underlie differences in ommatidia size and thus overall eye size between *D. mauritiana* and *D. simulans*. Our work provides new insights into ommatidia size regulation and the evolution of eye size. Together with evidence from other studies showing that changes in the timing of *ey* expression contributes to differences in ommatidia number in *Drosophila* [4], we have provided new insights into the genetic and developmental mechanisms that underlie the large diversity in *Drosophila* eye size [4, 19, 23, 25, 29–34]. Moreover, this evidence suggests that changes in the temporal expression of upstream transcription factors could be a widespread mechanism for morphological evolution.

## Methods

### Fly stocks and clonal analysis

*D. simulans* strain *yellow* (*y*), *vermillion* (*v*), *forked* (*f*) was obtained from the *Drosophila* Species Stock Center, San Diego, California (Stock no.14021–0251.146). *D. mauritiana* TAM16 is a wild-type inbred strain [29]. *UAS-miR-otd* and *UAS-otd* (III) were kindly provided by Henry Sun [47]. *GMR-GAL4* [68] was used to drive expression of the transgenes. To generate mitotic clones of mutant *otd* in developing eyes, we used *otd[YH13]*, *neoFRT19A/FM7c* and *RFP*, *neoFRT19A; ey-Flp*, which were obtained from Bloomington Stock Centre (#8675 and #67173 respectively). The *ato3’FL-GAL4* is described in [47] and drives expression in the MF and early photoreceptors (Additional File 11: Fig. S6). The *UAS-otd* line is the stock with Flybase number FBst0005541 (P(UAS-oc.F)RF1, w1118). The *UAS-GFP* line was like in [69]. To generate *UAS-otd;; ato3’FL-GAL4* flies (“*ato* > *otd”*) we crossed *UAS-otd* females to *ato3’FL-GAL4/*TM6B males. As controls, we used *UAS-otd;;TM6B* siblings (“*otd*, + *”*) and the parental *ato3’FL-GAL4/*TM6B strain (*“ato* > + *”*). To determine the area of ommatidia, we used females. Heads were mounted and photographed as in [70]. We measured the area of three strips of 3X5 facets using the “polygonal tool” of ImageJ from the anterior-ventral region (adjacent to the second and third antennal segments) of the two eyes and then the values were averaged. The average area/15 was the facet area of each individual. This was carried out for the experimental genotype (*UAS-otd;; ato3’FL-GAL4* flies) and the two controls (*UAS-otd;;TM6B* and *ato3’FL-GAL4/*TM6B). We performed a one-way ANOVA to test differences between the three different samples. We also carried out a Tukey's HSD to identify which specific groups differed significantly from each other.

*otd* mutant clones were induced in developing eyes using the Flp/FRT system [71]. Female flies of the genotype *otd[YH13]*, *neoFRT19A/FM7c* were crossed with males of the genotype *RFP*, *neoFRT19A; ey-Flp*. Female F1 progeny were examined for the lack of the FM7c balancer and these flies were prepared for SEM analysis.

### Synchrotron radiation microtomography

Fly heads were removed from the body and placed into fixative (2% PFA, 2.5% GA in 0.1 M sodium cacodylate buffer overnight at 4 °C. Heads were washed in water, then placed into 1% osmium tetroxide for 48 h at 4 °C, then washed and dehydrated in increasing concentrations of ethanol up to 100%. Heads were then infiltrated with increasing 812 Epon resin concentrations up to 100% over 5 days and polymerised in embedding moulds for 24 h at 70 °C.

Heads were scanned at the TOMCAT beamline of the Swiss Light Source (Paul Scherrer Institute, Switzerland [72]). Scans were performed using a 16 keV monochromatic beam with a 20 μm LuAG:Ce scintillator. Resin blocks were trimmed and mounted using soft wax and scanned using × 20 combined magnification (effective pixel size 325 nm) and a propagation distance of 25 mm. Two thousand projections were taken as the heads rotated through 180°, each with 200 ms exposure. Projections were reconstructed into 8-bit tiff stacks and Paganin filtered (delta = 1^−8^, beta = 2^−9^ [73] using custom in-house software [74]. Tiff stacks were segmented in Amira (v2019.2, Thermo Fisher) for measurements of facet area.

### SEM microscopy

Fly heads were fixed in Bouin’s for 2 h. Then, 1/3 of total volume was replaced by 100% ethanol to fully immerse heads in Bouin’s and were left to fix overnight. Heads were washed and dehydrated 2 × 70% ethanol overnight, 2 × in 100% ethanol and finally critical point dried and mounted onto sticky carbon tabs on SEM stubs, gold coated and imaged in a Hitachi S-3400N SEM with secondary electrons at 5 kV.

### Markers and introgression lines

Previously, we generated three replicate introgression lines (IL1, IL3 and IL4) by introgressing the region between y (X:178,985) and v (X:10,264,389, Supplementary Table S2b) from *D. mauritiana* TAM16 into *D. simulans y*,* v*,* f* [29]. Males recombinant within the introgressed region (with phenotypes: *y*, *f* or *v*, *f*) from the 3 replicate lines were collected at backross 7. These individuals were genotyped with eleven new additional markers (Additional File 2: Table S2).

Significant association between each marker and eye size was tested (*F*-test, type III sum of squares SS) by performing a single-marker ANOVA on the residuals of eye area regressed onto T1 tibia length for each replicate (introgression line (IL 1,3 and 4; *n* = 20–60, Additional File 2: Table S2). Multiple testing was corrected using Bonferroni correction. All ANOVA models were fitted in the R statistical environment (R Development Core Team 2012) using the CAR package [75].

To narrow down the 2 Mb region, the X chromosome region between *y* and *v* from *D. mauritiana* TAM16 was re-introgressed into *D. simulans y*,* v*,* f* (as in [29]) and *y*,* f* females were backcrossed from multiple replicate lines to *y*,* v*,* f* males for a further nine generations. At the end of the egg-laying cycle of that generation, we collected mothers and genotyped them for molecular markers located in the 2 Mb region (Additional File 2: Table S2). Four mothers with breakpoints within this region were identified. Two of them were siblings (IL9.1a and IL9.1b) and they had the same 4th great-grandmother as IL9.3 and the same 7th great-grandmother as IL9.2. Male progeny available for each of these females was collected and genotyped and phenotyped for eye area, ommatidia diameter, ommatidia number and T1 tibia length as described previously in [23] (Additional File 2: Table S2). To determine if the *D. mauritiana* DNA in the 2 Mb region resulted in larger eyes and larger ommatidia, *y*, *f* males (i.e. with some *D. mauritiana* DNA in the 2 Mb interval) were compared to that of their *y*,* v*,* f* sibling males (i.e. without *D. mauritiana* DNA) for each introgression line using one-tailed, two-sample, equal-variance *t*-tests.

### In situ* hybridisation and immunohistochemistry*

In situ hybridizations were carried out using a standard protocol with DIG-labelled antisense RNA probes. EADs were dissected and fixed at 120 h AEL for 40 min in 4% formaldehyde. To be able to compare the expression patterns avoiding technical differences (i.e. probe affinity and probe concentration), we first aligned the sequences from *D. mauritiana* and *D. simulans* and designed primers to generate RNA probes within fragments with at least 95% of similarity between them (Additional File 22: Table S10). This design allowed us to perform the in situ hybridization experiments using the same specific probes for each of the candidate genes at the same concentration for both species. The nitro blue tetrazolium/5-bromo-4-chloro-3′-indolyphosphate (NBT-BCIP) reaction was stopped at the same time. Candidate gene sequences were cloned into a TOPO PCR4 (*spirit*, *otd*, *Ppt1*, *CG1632*, *Es2* and *CG12112*) or pCRII (*CG1885*, *Sptr*) vectors (Invitrogen) using specific primer pairs (Additional File 22: Table S10), respectively, following the manufacturer’s protocol. M13 forward and reverse primers were used to linearise the DNA. According to the vector and orientation of the fragments T3, T7 or SP6 RNA polymerase were used to generate the DIG-labelled riboprobes.

Immunostainings with Rabbit anti-Otd [44] and rat anti-Elav (7E8A10, Hybridoma bank) were performed at 1:1500 and 1:100 dilutions respectively using standard protocols, followed by anti-rat-Cy3 (Jackson Immuno Research) and anti-rabbit-Alexafluor 647 (Molecular probes) secondary AB staining, at 1:200. The actin cytoskeleton was stained with Alexafluor 488-Phalloidin (Molecular Probes) at 1:40 dilution for 30 min after secondary antibody incubation. Discs were mounted in Prolong Gold antifade reagent, supplemented with DAPI (Molecular Probes), and captured with a Zeiss LSM 510 confocal microscope. Images were processed using NIH ImageJ software. We used an Analysis of Covariance (ANCOVA) to test for differences between strains for Otd-positive ommatidia while adjusting for differences in development stage by using the number of ommatidial rows as a proxy for the latter. The ANCOVA was performed using base R v4.0.2 (R Core Team, 2020).

### RNA-seq

Flies were raised at 25 °C with a 12 h:12 h dark:light cycle and their eggs were collected in 2 h time periods. Freshly hatched L1 larvae were transferred into fresh vials in density-controlled conditions (30 freshly hatched L1 larvae per vial). EADs were dissected at three different developmental time points: 72, 96 and 120 h AEL and stored in RNALater (Qiagen, Venlo, Netherlands). Three biological replicates for each sample were generated. Total RNA was isolated using RNeasy Mini Kit (Qiagen). RNA quality was determined using the Agilent 2100 Bioanalyzer (Agilent Technologies, Santa Clara, CA, USA) microfluidic electrophoresis.

Library preparation for RNA-seq was performed using the TruSeq RNA Sample Preparation Kit (Illumina, catalog ID RS-122–2002) starting from 500 ng of total RNA. Accurate quantitation of cDNA libraries was performed using the QuantiFluor™dsDNA System (Promega, Madison, Wisconsin, USA). The size range of final cDNA libraries was determined by applying the DNA 1000 chip on the Bioanalyzer 2100 from Agilent (280 bp). cDNA libraries were amplified and sequenced using cBot and HiSeq 2000 (Illumina): only 120 h EAD samples were sequenced as paired-end (PE) reads (2 × 100 bp), all the other samples were sequenced in single-end (SE) reads (1 × 50 bp). Sequence images were transformed to bcl files using the software BaseCaller (Illumina). The bcl files were demultiplexed to fastq files with CASAVA (version 1.8.2).

Quality control analysis using FastQC software (version 0.10.1, Babraham Bioinformatics) was performed. All RNAseq reads are accessible in the Short Read Archive through umbrella BioProject PRJNA666691 (containing PRJNA374838 and PRJNA666524). To allow using the same mapping parameters for all samples, PE 100 bp reads were converted into SE 50 bp by splitting the reads in half and merging right and left reads into a single file prior to read mapping.

The reciprocally re-annotated references described in [41] were used to map the species-specific reads. Bowtie2 [76] was used to map the reads to each reference (–very-sensitive-local –N 1), and the idxstats command from SAMtools v0.1.19 [77] was used to summarise the number of mapped reads. HTSFilter [78] was used with default parameters to filter out genes with very low expression in all samples. For the remaining genes in each pair-wise comparison, differential expression was calculated using DESeq2 v1.2.7. with default parameters [79].

### ATAC-seq library preparation and sequencing

Samples were obtained following the same procedure as for the RNA-seq experiments: flies were raised at 25 °C with a 12 h:12 h dark:light cycle. Freshly hatched L1 larvae were transferred into vials with density-controlled conditions. EADs were dissected at 96 and 120 h AEL and maintained in ice cold PBS. Imaginal disc cells were lysed in 50 μl Lysis Buffer (10 mM Tris–HCl, pH = 7.5; 10 mM NaCl; 3 mM MgCl_2_; 0.1% IGEPAL). Nuclei were collected by centrifugation at 500 g for 5 min. 75,000 nuclei were suspended in 50 μl Tagmentation Mix [25 μl Buffer (20 mM Tris- CH_3_COO^−^, pH = 7.6; 10 mM MgCl_2_; 20% Dimethylformamide); 2.5 μl Tn5 Transposase; 22.5 μl H_2_O] and incubated at 37 °C for 30 min. After addition of 3 μl 2 M NaAC, pH = 5.2 DNA was purified using a QIAGEN MinElute Kit. PCR amplification for library preparation was done for 14 cycles with NEBNext High Fidelity Kit; primers were used according to [48]. Paired end 50 bp sequencing was carried out by the Transcriptome and Genome Analysis Laboratory Goettingen, Germany.

### ATAC-seq peak calling and differential binding site analysis

ATAC-seq raw reads were generated from the following samples (2 replicates each): *D. simulans* larvae at 96 and 120 hAEL and *D. mauritiana* larvae at 96 and 120 hAEL. These reads were mapped to strain-specific genomes of *D. mauritiana* and *D. simulans* [41] using Bowtie2 (version 2.3.4.1) [76] with the parameter –X2000. The Samtools suite v0.1.19 [77] was used to convert *.sam to *.bam files and to further process the mapped reads. Duplicates were removed using Picard (version 2.20.2) with the parameter REMOVE_DUPLICATE = TRUE. Bam files were then converted to bed files using the Bedtools (version 2.24) bamtobed command. Reads were centred according to [80]. These reads were then converted to the *D. simulans* or *D. mauritiana* coordinate system using liftOver (1.14.0) with custom prepared chain files, one for the conversion of *D. mauritiana* coordinates to *D. simulans* coordinates and one for the conversion of *D. simulans* coordinates to *D. mauritiana* coordinates. Peaks were then called using MACS2 (version 2.1.2, [81]) with the following parameters: –shift – 100, extsize 200, -q 0.01.

We used the Diffbind package (version 2.12.0, [82]) in R (version 3.6.1.) to search for differentially accessible ATAC-seq regions. A consensus peak set of 19,872 peaks (96 h AEL) and 15,868 peaks (120 h AEL) was used for all samples and the reads were counted for each identified peak with the dba.count command. For each time point separately we used the dba.analyze command with default parameters to get differentially accessible peaks between the two species. This command uses by default the DESeq2 analysis. All plots were generated with the DiffBind package.

### Reporter assays

All APREs with the exception of *D. mauritiana-APRE6*, *D. simulans-APRE11* and *D. mauritiana-APRE11* were cloned upstream of GAL4 into the pBPGUw backbone (a gift from Gerald Rubin, Addgene plasmid 17,575) by Genewiz. *D. mauritiana-APRE6*, *D. simulans-APRE11 and D. mauritiana-APRE11* were first sub-cloned into the pENTR-TOPO-D plasmid backbone then shuttled into the pBPGUw backbone using Gateway cloning. Sequences of all APREs used can be found in Additional File 23: Table S11.

*otd-APRE7-8-GAL4* lines with *D. mauritiana* and *D. simulans* sequences were crossed with *UAS-nlsGFP* to characterise the activity of APRE7-8. EADs were dissected and fixed according to standard protocols. Immunostaining was performed with mouse anti-Elav (Hybridoma Bank) at 1:100 and rabbit anti-GFP (Molecular probes) at 1:1000, followed by secondary antibody incubation with anti-rabbit Alexa Fluor 488 and anti-mouse Alexa Fluor 568, both at 1:400. Imaging was performed using a Leica Stellaris microscope with a 40X objective and zoom 2. Images were processed using the NIH ImageJ software.

We used Elav staining to count the number of ommatidial rows as a measure of developmental stage. We define the first row showing full enhancer activity as the first row counting from the posterior margin of the disc with more than 90% of the ommatidia positive for enhancer signal/GFP signal. For all discs, we determined the developmental time and the first row with full enhancer activity. Then, we calculated the distance between the MF and the first row of full enhancer activity as the difference between the total ommatidial rows and the first ommatidial row with full enhancer activity.

We then plotted the first row with full enhancer activity at different developmental times and fitted a regression line to model the observed linear trend for each species, with calculated *R* value for correlation and *p* value of fit.

Violin plots were used to show the distribution of distances between the morphogenetic furrow and the first row of full enhancer activity. We used Welch’s *t*-test to test whether the difference in distances between species was significant. In the case of the three-species comparison (Additional File 18: Fig. S10), we use the nonparametric Games-Howell test to make pairwise comparisons among the three species. We used an analysis of covariance (ANCOVA) to test for differences between strains for Otd-positive ommatidia rows while adjusting for differences in development stage by using the number of ommatidial rows as a proxy for the latter. The ANCOVA was performed using base R v4.0.2 (R Core Team, 2020).

### Prediction of transcription factor binding sites and potential target genes

To search for TFBM of potential *otd* regulators, we used the universalmotif package (version 1.20.0) using its function to scan sequences. The JASPAR core and CISBP databases were used for screening DNA sequences of ATAC-seq peaks in the *otd* APRE7-8 sequence with all possible TFBSs from *Drosophila* with a threshold of *p*-value > 0.001. Potential fixed changes in the 2.5 kb APRE7-8 sequence of *D. mauritiana* were inferred from aligning the sequences of this region from strains of *D. mauritiana* (Red3, mau12, mav2 and TAM16), *D. simulans* (m3 and w501) and the *D. melanogaster* sequence using Clustal Omega with default parameters [77].

To define a list of potential Otd target genes, we used an Otd-motif (Dmelanogaster-FlyFactorSurvey-Oc_Cell_FBgn0004102) from the MotifDB package (version 1.16.1), which provides a collection of available transcription factors in R (version 3.3.3). We searched for Otd binding sites in accessible chromatin regions with the findMotifsGenome.pl command implemented in the HOMER (version V4.10.4, [83]) in all samples. All peaks with a predicted Otd motif were annotated to an associated gene using the annotatePeaks.pl command by HOMER and combined all time points and both species into one file. We then looked for the number of genes with an annotated Otd motif and found 1,148 unique genes, which we overlapped with our RNA-seq dataset to find out which of these target genes were differentially expressed between the two species. GO term enrichment analysis of putative Otd target genes was performed using the online tool Metascape [84].

We used the online STRING database that integrates all known and predicted associations between proteins based on evidence from a variety of sources [85], to construct networks of DEG encoded proteins. To visualize the network and map genes/prot with Otd motifs, we applied the Cytoscape software [86].

## Supplementary Information

Additional file 1: Table S1. Measurements of ommatidia size of *D. mauritiana *and *D. simulans *eyes.

Additional file 2: Table S2. Mapping and introgression data.

Additional file 3: Fig. S1. Ommatidia number and body size of the IL lines. Ommatidia number (left) and T1 Tibia length (right) was not significantly different between *y*, *f *males and their *y*,* v*,* f* sibling males for each introgression line (two-tailed, two-sample, equal-variance t-tests).

Additional file 4: Fig. S2. RNA-seq datasets. (a) Heat-map of all RNA-seq samples. (b) PCA plot of all RNA-seq samples.

Additional file 5: Table S3. Pair-wise differential gene expression analysis (D.sim vs. D.mau) results for each time point (72 hAEL, 96 hAEL and 120 hAEL).

Additional file 6: Fig. S3. *otd *expression in 120 hAEL eye imaginal discs. (a) *D. mauritiana *EAD at 120h. Intensity plots show *otd *expression intensity measured in the red square area with ImageJ plot intensity tool. (b) *D. simulans *EAD at 120h. (c) Table and plot showing the area under the curve for each of the discs in which the intensity of *otd *signal was measured. Black arrowhead: Morphogenetic furrow; oc: ocellar region; dev eye: eye region.

Additional file 7: Table S4. Measurements of plot intensities of *otd* in situ hybridization in *D. mauritiana *and *D. simulans *eye discs.

Additional file 8: Fig. S4. *otd *expression in 110 hAEL eye imaginal discs. (a) *D. mauritiana *EAD at 110h. (b) *D. simulans *EAD at 110 hAEL. Red arrowheads highlight expression of *otd *in some *D. mauritiana *discs. Black arrowhead: Morphogenetic furrow; oc: ocellar region; dev eye: eye region.

Additional file 9: Table S5. Measurements of Otd-positive ommatidia in *D. mauritiana *and *D. simulans *eye discs

Additional file 10: Fig. S5. *otd *expression in pupal eyes. (a) *D. mauritiana *pupal eye (48 h APF) stained with Phalloidin (Actin, a’), anti-Elav marks photoreceptors (a”) and Otd is expressed in all ommatidia (a”’). (b) Otd is present in all ommatidia in *D. simulans *pupal eyes, (b’) Actin (Phalloidin) highlights ommatidia area, (b”) anti-Elav marks photoreceptors and Otd protein is shown in b”’.

Additional file 11: Fig. S6. Effects of loss and gain of *otd *expression in ommatidial structure and facet size in *D. melanogaster*. Loss of *otd *causes defects in ommatidia structure. (a-c) Knockdown of *otd *by expressing *UAS-miR-otd *with *GMR-GAL4 *driver in *D. melanogaster* eye. (a-a’) *GMR-GAL. *(b-b’) *GMR-GAL/ UAS-miR-otd *shows a rough eye phenotype due to defects in ommatidia arrangements*. *(c-c’) *GMR-GAL/ UAS-miR-otd *phenotype is rescued by co-expressing *UAS-otd*. (d-d’) *otd i*mutant mitotic clones also show defects in ommatidia size and organisation. (e) Comparison of facet area from eyes of *UAS-otd (“+*,* otd”)*, *ato3’FL-GAL4 (“ato>+) *and *UAS-otd; ato3’FL-GAL4 (“ato>otd”) *female adults. Facet area is significantly larger in the experimental genotype (*“ato>otd”*) than in the two control genotypes. See main text for details.

Additional file 12: Table S6. *ato-Gal4> UAS-otd* ommatidia size.

Additional file 13: Fig. S7. Alignment of *D. mauritiana* TAM16 and *D. simulans* *y*, *v*, *f* Otd protein sequences.

Additional file 14: Fig. S8. Topological Domain Associated with *otd *locus in *D. melanogaster *from http://chorogenome.ie-freiburg.mpg.de/.

Additional file 15: Table S7. Differential peak calling for *D. mau *and *D. sim *96 hAEL and 120 hAEL ATAC-seq data.

Additional file 16: Fig. S9. Activity of tested APREs in the *otd *locus of *D. simulans *and *D. mauritiana*. L3 instar eye-antenna imaginal discs from *D. melanogaster *lines containing *D. mauritiana-APRE4-GAL4*,* D. mauritiana-APRE6-GAL4*, *D. mauritiana-APRE7-8-GAL4*,* D. simulans-APRE11-GAL4*,* D. mauritiana-APRE13-14-GAL4*,* D. mauritiana-APRE16-17-GAL4*,* D. mauritiana-APRE19-GAL4* crossed to *UAS-GFP *and stained with DAPI (grey). Scale bar = 50μm.

Additional file 17: Table S8. TFBM in differential open chromatin regions in *otd *locus of *D. simulans *and *D. mauritiana*.

Additional file 18: Fig. S10. *D. melanogaster*, *D. simulans *and* D. mauritiana* *otd-APRE7-8 *enhancer activity in 3^rd^ instar larvae eye imaginal discs. (a) *D. melanogaster*, *D. simulans *and* D. mauritiana* *otd-APRE7-8* 3^rd^ instar larvae eye imaginal discs immunostained with anti-Elav antibody to visualise the progression of ommatidia maturation as proxy for the developmental time. (b) Plot showing the number of GFP-positive ommatidia rows (*x*-axis) activated by *otd-APRE7-8* at different developmental time points (*y*-axis, developmental points inferred by number of ommatidia rows) for both species. (c) Violin plots showing the distance between MF and the first row of *otd-APRE7-8* activity. *D. mauritiana otd-APRE7-8* is active earlier during the differentiation of ommatidia.

Additional file 19: Dataset S1. Clustal Omega alignment of the 2.5 kb APRE7-8 region from selected strains of *D. mauritiana*, *D. simulans* and *D. melanogaster* including the sequences used in enhancer reporter constructs for each species (*otd-APRE7-8*).

Additional file 20: Fig. S11. Schematic showing the position of the 2.5 kb APRE7-8 within the *otd* locus on the X chromosome of *D. melanogaster*. A 1.5 kb region shown to drive similar expression to the 2.5 kb region is indicated by a purple bar (37). Below is shown the ATAC-seq profile for this region and the positions of potential fixed mutations specific to *D. mauritiana* (arrows indicate SNPs and rectangles indicate short indels) with predicted binding sites for Otd, So and Cut in *D. melanogaster* indicated by arrows. Binding sites indicated with an asterisk contain mutations in *D. mauritiana*.

Additional file 21: Table S9. Differentially expressed genes with predicted Otd TFBMs in their associated open chromatin regions.

Additional file 22: Table S10. Primers used to generate probes for in situ hybridization.

Additional file 23: Table S11. Sequences used to generate reporter lines.

## Data Availability

All RNAseq and ATACseq reads are accessible in the Short Read Archive through umbrella BioProject PRJNA666691 [30] (https://www.ncbi.nlm.nih.gov/bioproject/?term=PRJNA666691, containing PRJNA374838 and PRJNA666524).
