# Supplementary material for: Heterochrony in orthodenticle expression is associated with ommatidial size variation between Drosophila species
Source: BMC Biol. 2025 Feb 4;23:34. doi: 10.1186/s12915-025-02136-8 (PMC11792340; doi:10.1186/s12915-025-02136-8)
Supplement: Supplementary file 3 — Additional file 3: Fig. S1. Ommatidia number and body size of the IL lines. Ommatidia number (left) and T1 Tibia length (right) was not significantly different between y, f males and their y, v, f sibling males for each introgression line (two-tailed, two-sample, equal-variance t-tests). [file 12915_2025_2136_MOESM3_ESM.pdf]

Figure S1

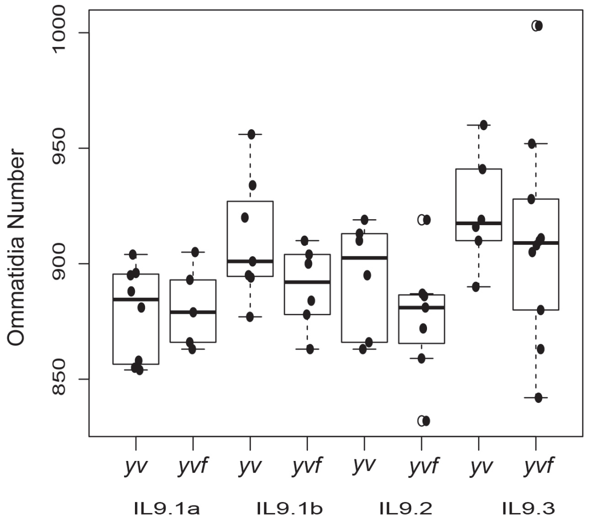

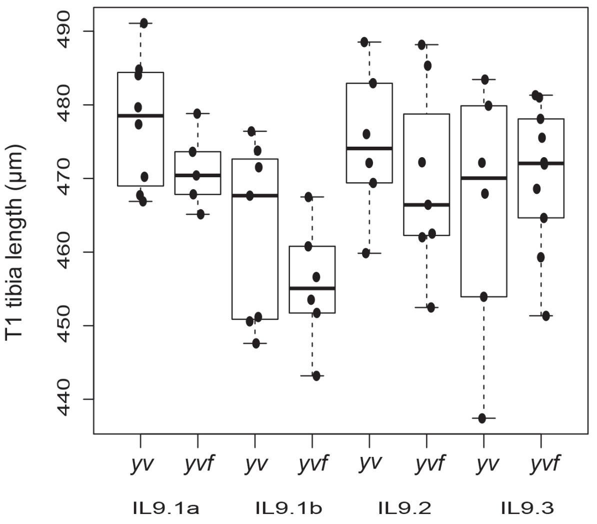

Figure S1. Ommatidia number and body size of the IL lines. Ommatidia number and body size did not differ between y, f males and their respective y, v, f sibling males.
