# Supplementary material for: Heterochrony in orthodenticle expression is associated with ommatidial size variation between Drosophila species
Source: BMC Biol. 2025 Feb 4;23:34. doi: 10.1186/s12915-025-02136-8 (PMC11792340; doi:10.1186/s12915-025-02136-8)
Supplement: Supplementary file 4 — Additional file 4: Fig. S2. RNA-seq datasets. (a) Heat-map of all RNA-seq samples. (b) PCA plot of all RNA-seq samples. [file 12915_2025_2136_MOESM4_ESM.pdf]

Figure S2

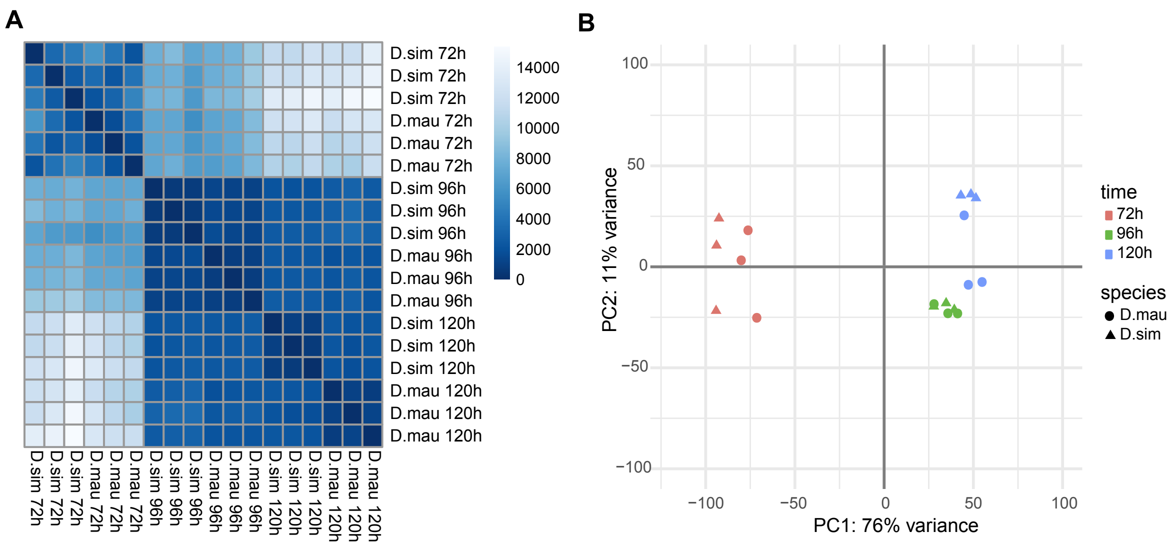

Figure S2. RNA-seq datasets. (a) Heat-map of all RNA-seq samples. **(b)** PCA plot of all RNA-seq samples
