# Supplementary material for: Heterochrony in orthodenticle expression is associated with ommatidial size variation between Drosophila species
Source: BMC Biol. 2025 Feb 4;23:34. doi: 10.1186/s12915-025-02136-8 (PMC11792340; doi:10.1186/s12915-025-02136-8)
Supplement: Supplementary file 6 — Additional file 6: Fig. S3. otd expression in 120 hAEL eye imaginal discs. (a) D. mauritiana EAD at 120h. Intensity plots show otd expression intensity measured in the red square area with ImageJ plot intensity tool. (b) D. simulans EAD at 120h. (c) Table and plot showing the area under the curve for each of the discs in which the intensity of otd signal was measured. Black arrowhead: Morphogenetic furrow; oc: ocellar region; dev eye: eye region. [file 12915_2025_2136_MOESM6_ESM.pdf]

Figure S3

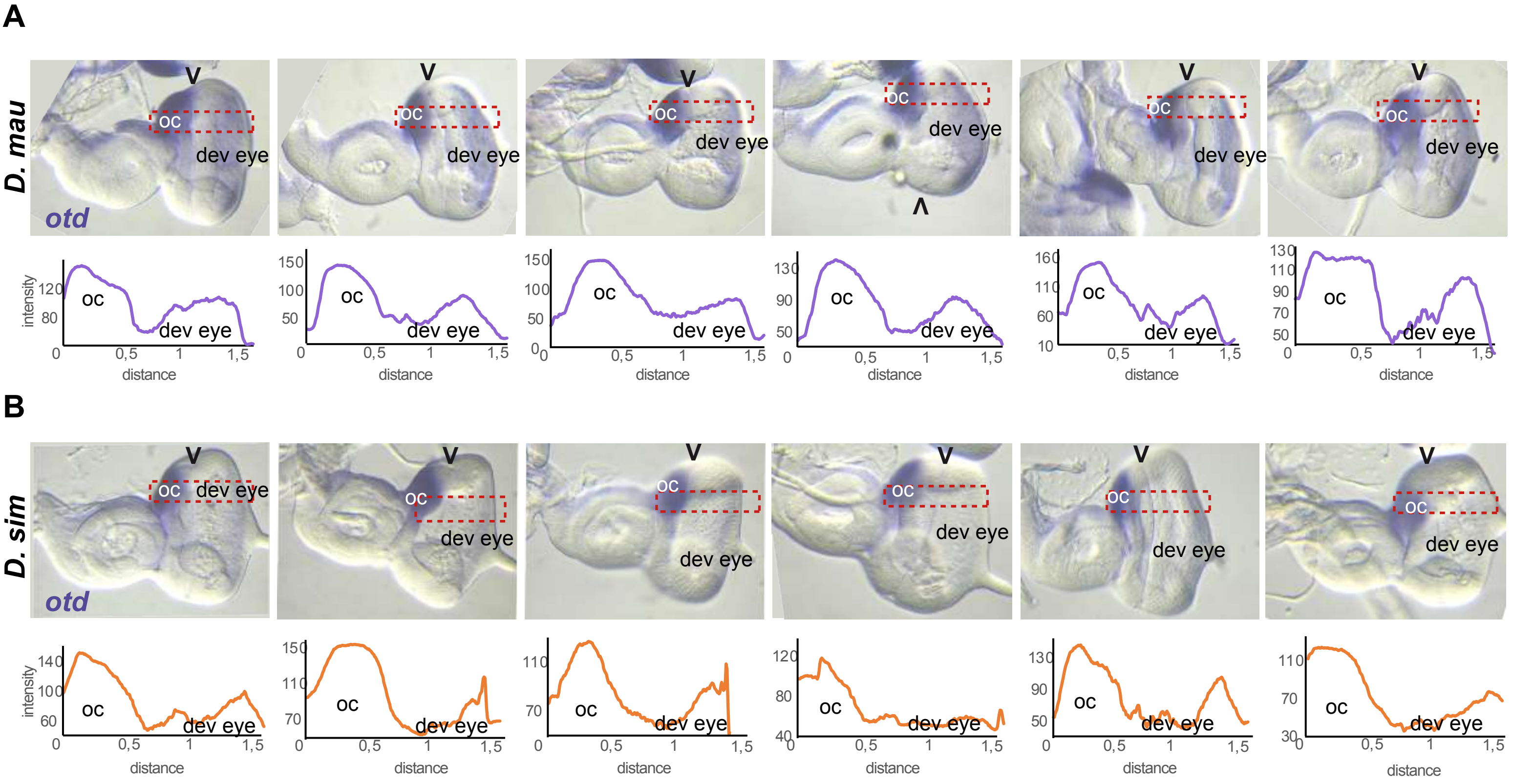

C

Species	Area
D. mau	37,22
D. mau	40,51
D. mau	38,08
D. mau	27,18
D. mau	38,5
D. mau	34,75
D. sim	20,22
D. sim	13,11
D. sim	16,88
D. sim	8,99
D. sim	22,23
D. sim	15,2

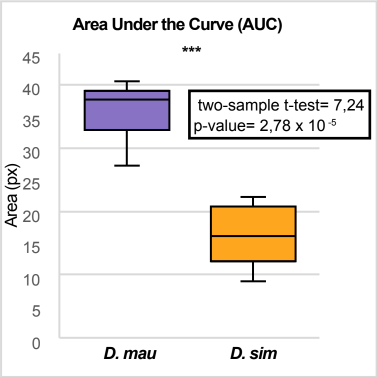

Figure S3. *otd* expression in 120h eye imaginal discs. (A) *D. mauritiana* AED at 120h. Intensity plots show *otd* expression intensity measured in the red square area with ImageJ plot intensity tool. **(B)** *D. simulans* AED at 120h. **(C)** Table and plot showing the area under the curve for each of the discs in which the intensity of *otd* signal was measured. Black arrowhead: Morphogenetic furrow; oc: ocellar region; dev eye: eye region.
