# Supplementary material for: Heterochrony in orthodenticle expression is associated with ommatidial size variation between Drosophila species
Source: BMC Biol. 2025 Feb 4;23:34. doi: 10.1186/s12915-025-02136-8 (PMC11792340; doi:10.1186/s12915-025-02136-8)
Supplement: Supplementary file 8 — Additional file 8: Fig. S4. otd expression in 110 hAEL eye imaginal discs. (a) D. mauritiana EAD at 110h. (b) D. simulans EAD at 110 hAEL. Red arrowheads highlight expression of otd in some D. mauritiana discs. Black arrowhead: Morphogenetic furrow; oc: ocellar region; dev eye: eye region. [file 12915_2025_2136_MOESM8_ESM.pdf]

Figure S4

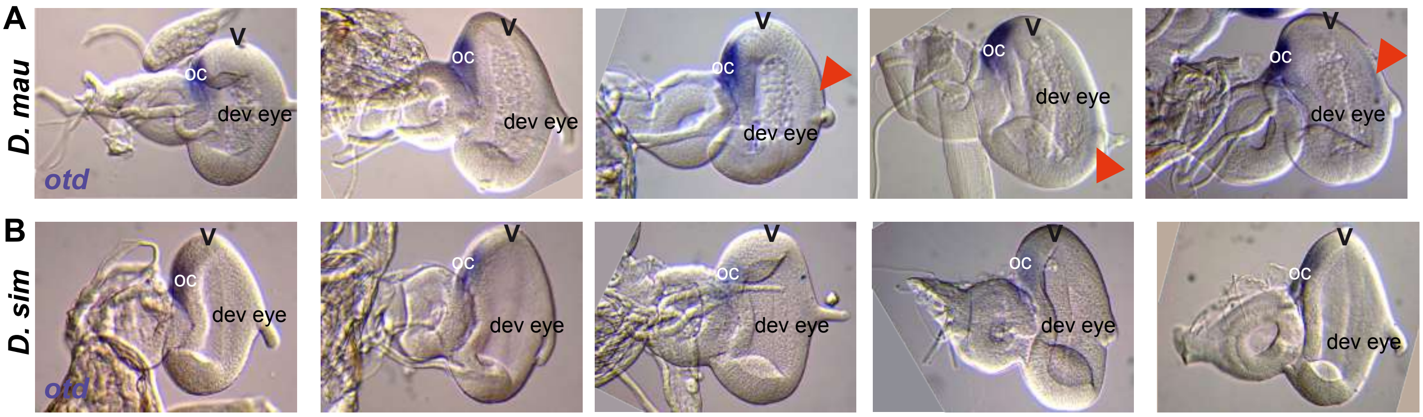

Figure S4. *otd* expression in 110h eye imaginal discs. (A) *D. mauritiana* AED at 110h. **(B)** *D. simulans* AED at 110h. Red arrowheads highlight expression of *otd* in some *D. mauritiana* discs. Black arrowhead: Morphogenetic furrow; oc: ocellar region; dev eye: eye region.
