# Supplementary material for: Heterochrony in orthodenticle expression is associated with ommatidial size variation between Drosophila species
Source: BMC Biol. 2025 Feb 4;23:34. doi: 10.1186/s12915-025-02136-8 (PMC11792340; doi:10.1186/s12915-025-02136-8)
Supplement: Supplementary file 10 — Additional file 10: Fig. S5. otd expression in pupal eyes. (a) D. mauritiana pupal eye (48 h APF) stained with Phalloidin (Actin, a’), anti-Elav marks photoreceptors (a”) and Otd is expressed in all ommatidia (a”’). (b) Otd is present in all ommatidia in D. simulans pupal eyes, (b’) Actin (Phalloidin) highlights ommatidia area, (b”) anti-Elav marks photoreceptors and Otd protein is shown in b”’. [file 12915_2025_2136_MOESM10_ESM.pdf]

Figure S5

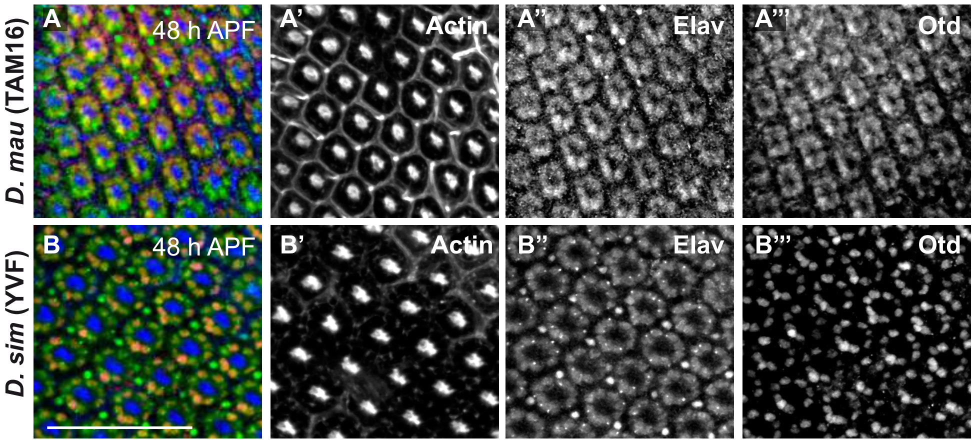

Figure S5. Otd expression in pupal eyes. **(A)** *D. mauritiana* pupal eye (48 h APF) stained with Phalloidin (Actin, a'), anti-Elav marks photoreceptors **(A'')** and Otd is expressed in all ommatidia **(A'''). (B)** Otd is present in all ommatidia in *D. simulans* pupal eyes, **(B')** Actin (Phalloidin) highlights ommatidia area, **(B'')** anti-Elav marks photoreceptors and Otd protein is shown in **B'''**.
