# Supplementary material for: Heterochrony in orthodenticle expression is associated with ommatidial size variation between Drosophila species
Source: BMC Biol. 2025 Feb 4;23:34. doi: 10.1186/s12915-025-02136-8 (PMC11792340; doi:10.1186/s12915-025-02136-8)
Supplement: Supplementary file 11 — Additional file 11: Fig. S6. Effects of loss and gain of otd expression in ommatidial structure and facet size in D. melanogaster. Loss of otd causes defects in ommatidia structure. (a-c) Knockdown of otd by expressing UAS-miR-otd with GMR-GAL4 driver in D. melanogaster eye. (a-a’) GMR-GAL. (b-b’) GMR-GAL/ UAS-miR-otd shows a rough eye phenotype due to defects in ommatidia arrangements. (c-c’) GMR-GAL/ UAS-miR-otd phenotype is rescued by co-expressing UAS-otd. (d-d’) otd imutant mitotic clones also show defects in ommatidia size and organisation. (e) Comparison of facet area from eyes of UAS-otd (“+, otd”), ato3’FL-GAL4 (“ato>+) and UAS-otd; ato3’FL-GAL4 (“ato>otd”) female adults. Facet area is significantly larger in the experimental genotype (“ato>otd”) than in the two control genotypes. See main text for details. [file 12915_2025_2136_MOESM11_ESM.pdf]

Figure S6

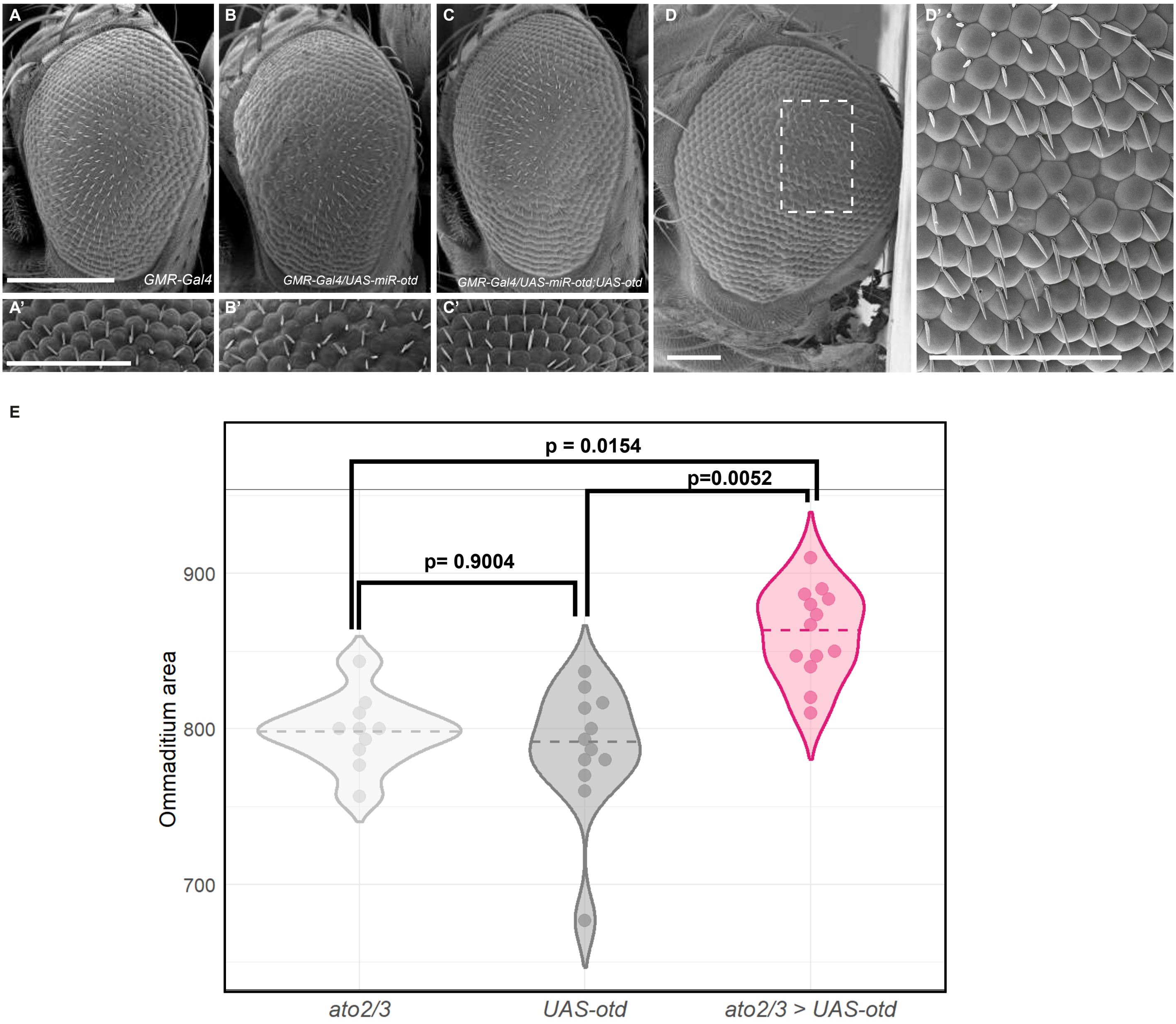

Figure S6. Loss and gain of *otd* causes defects in ommatidia structure.
(A-C) Knockdown of otd by expressing *UAS-miR-otd* with *GMR-GAL4* driver in *D. melanogaster* eye. **(A-A')** *GMR-GAL4.* **(B-B')** *GMR-GAL4/ UAS-miR-otd* shows a rough eye phenotype due to defects in ommatidia arrangements. **(C-C')** *GMR-GAL4/ UAS-miR-otd* phenotype is rescued by co-expressing *UAS-otd*. **(D-D')** *otd* mutant mitotic clones also show defects in ommatidia size and organisation. **(E)** Overexpression of otd with ato-Gal4 increases ommatidia area. One-way ANOVA test, F: 7,95 p-value: 0,0015; Tukey's HSD (p = 0.0154, p=0.0052,p=0.9004)
