# Supplementary material for: Heterochrony in orthodenticle expression is associated with ommatidial size variation between Drosophila species
Source: BMC Biol. 2025 Feb 4;23:34. doi: 10.1186/s12915-025-02136-8 (PMC11792340; doi:10.1186/s12915-025-02136-8)
Supplement: Supplementary file 13 — Additional file 13: Fig. S7. Alignment of D. mauritiana TAM16 and D. simulans y, v, f Otd protein sequences. [file 12915_2025_2136_MOESM13_ESM.pdf]

Figure S7

```
CLUSTAL O(1.2.4) multiple sequence alignment


Dmau_TAM16      MAAGFLKSGDLGPHPHSYGGPHPHHSVPHGPLPPGMPMPSLGPFGLPHGLEAVGFSQGVN 60
Dsim            MAAGFLKSGDLGPHPHSYGGPHPHHSVPHGPLPPGMPMPSLGPFGLPHGLEAVGFSQGVN 60
Dmau            MAAGFLKSGDLGPHPHSYGGPHPHHSVPHGPLPPGMPMPSLGPFGLPHGLEAVGFSQGVN 60
                ************************************************************
                                       Homeodomain
Dmau_TAM16      TRKQRRERTTFTRAQLDVLEALFGKTRYPDIFMREEVALKINLPESRVQVWFKNRRAKCR 120
Dsim            TRKQRRERTTFTRAQLDVLEALFGKTRYPDIFMREEVALKINLPESRVQVWFKNRRAKCR 120
Dmau            TRKQRRERTTFTRAQLDVLEALFGKTRYPDIFMREEVALKINLPESRVQVWFKNRRAKCR 120
                ************************************************************

Dmau_TAM16      QQLQQQQQSNSLSSSKNASGGGSGGSCSSSSANSRSNSNNNGSSSNNNSQSSGGNNSNKS 180
Dsim            QQLQQQQQSNSLSSSKNASGGGSGGSCSSSSANSRSNSNNNGSSSNNNSQSSGGNNSNKS 180
Dmau            QQLQQQQQSNSLSSSKNASGGGSGGSCSSSSANSRSNSNNNGSSSNNNSQSSGGNNSNKS 180
                ************************************************************

Dmau_TAM16      SQKQGNSQSSQQGGGSSGGNNSNNNSAAAAASAAAAVAAAQSIKTHHSSFLSAAAAAASG 240
Dsim            SQKQGNSQSSQQGGGSSGGNNSNNNSAAAAASAAAAVAAAQSIKTHHSSFLSAAAAAASG 240
Dmau            SQKQGNSQSSQQGGGSSGGNNSNNNSAAAAASAAAAVAAAQSIKTHHSSFLSAAAAAASG 240
                ************************************************************

Dmau_TAM16      GTNQSANNNSNNNNQGNSTPNSSSSGGGGGSQAGGHLSAAAAAASLNVTAAHQNSSPLLP 300
Dsim            GTNQSANNNSNNNNQGNSTPNSSSSGGGGGSQAGGHLSAAAAAAALNVTAAHQNSSPLLP 300
Dmau            GTNQSANNNSNNNNQGNSTPNSSSSGGGGGSQAGGHLSAAAAAAALNVTAAHQNSSPLLP 300
                ********************************************:***************

Dmau_TAM16      TPATSVSPVSIVCKKEHLSGGYGSSVGGGGXXXXXXXXXXXXXXXXXXXXXXVGVGVSQD 360
Dsim            TPATSVSPVSIVCKKEHLSGGYGSSVGGGGGGGGGASSGGLNLGVGVGVGVGVGVGVSQD 360
Dmau            TPATSVSPVSIVCKKEHLSGGYGSSVGGGGGGGGGASSGGLNLGVGVGVGVGVGVGVSQD 360
                ******************************                      ********

Dmau_TAM16      LLRSPYDQLKDAGGDIGAGVHHHHSIYXXAAGSNPRLLQPGGNITPMDSSSSITTPSPPI 420
Dsim            LLRSPYDQLKDAGGDIGAGVHHHHSIYGSAAGSNPRLLQPGGNITPMDSSSSITTPSPPI 420
Dmau            LLRSPYDQLKDAGGDIGAGVHHHHSIYGSAAGSNPRLLQPGGNITPMDSSSSITTPSPPI 420
                ***************************  *******************************

Dmau_TAM16      TPMSPQSAAAAAHAAQSAQSAHHSAAHSAAYMSNHDSYNFWHNQYQQYPNNYAQAPSYYS 480
Dsim            TPMSPQSAAAAAHAAQSAQSAHHSAAHSAAYMSNHDSYNFWHNQYQQYPNNYAQAPSYYS 480
Dmau            TPMSPQSAAAAAHAAQSAQSAHHSAAHSAAYMSNHDSYNFWHNQYQQYPNNYAQAPSYYS 480
                ************************************************************

Dmau_TAM16      QMEYFSNQNQVNYNMGHSGYTASNFGLSPSPSFTGTVSAQAFSQNSLDYMSPQDKYANMV 540
Dsim            QMEYFSNQNQVNYNMGHSGYTASNFGLSPSPSFTGTVSAQAFSQNSLDYMSPQDKYANMV 540
Dmau            QMEYFSNQNQVNYNMGHSGYTASNFGLSPSPSFTGTVSAQAFSQNSLDYMSPQDKYANMV 540
                ************************************************************
```

Figure S7. Alignment of *D. mauritiana TAM16* and *D. simulans y, v, f* Otd protein sequences
