# Supplementary material for: Heterochrony in orthodenticle expression is associated with ommatidial size variation between Drosophila species
Source: BMC Biol. 2025 Feb 4;23:34. doi: 10.1186/s12915-025-02136-8 (PMC11792340; doi:10.1186/s12915-025-02136-8)
Supplement: Supplementary file 14 — Additional file 14: Fig. S8. Topological Domain Associated with otd locus in D. melanogaster from http://chorogenome.ie-freiburg.mpg.de/. [file 12915_2025_2136_MOESM14_ESM.pdf]

Figure S8

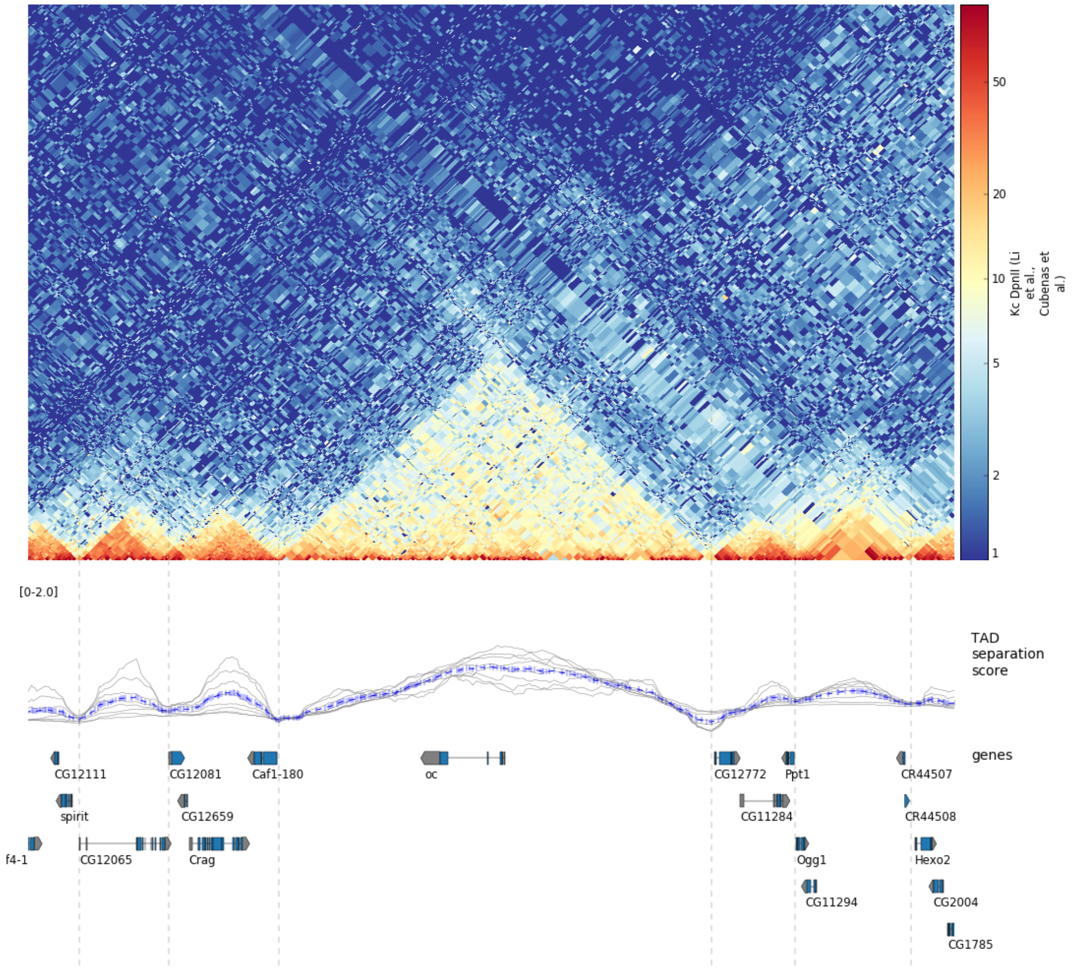

Figure S7. Topological Domain Associated with otd locus in *D. melanogaster* from http://chorogenome.ie-freiburg.mpg.de/
