# Supplementary material for: Heterochrony in orthodenticle expression is associated with ommatidial size variation between Drosophila species
Source: BMC Biol. 2025 Feb 4;23:34. doi: 10.1186/s12915-025-02136-8 (PMC11792340; doi:10.1186/s12915-025-02136-8)
Supplement: Supplementary file 16 — Additional file 16: Fig. S9. Activity of tested APREs in the otd locus of D. simulans and D. mauritiana. L3 instar eye-antenna imaginal discs from D. melanogaster lines containing D. mauritiana-APRE4-GAL4, D. mauritiana-APRE6-GAL4, D. mauritiana-APRE7-8-GAL4, D. simulans-APRE11-GAL4, D. mauritiana-APRE13-14-GAL4, D. mauritiana-APRE16-17-GAL4, D. mauritiana-APRE19-GAL4 crossed to UAS-GFP and stained with DAPI (grey). Scale bar = 50μm. [file 12915_2025_2136_MOESM16_ESM.pdf]

Figure S9

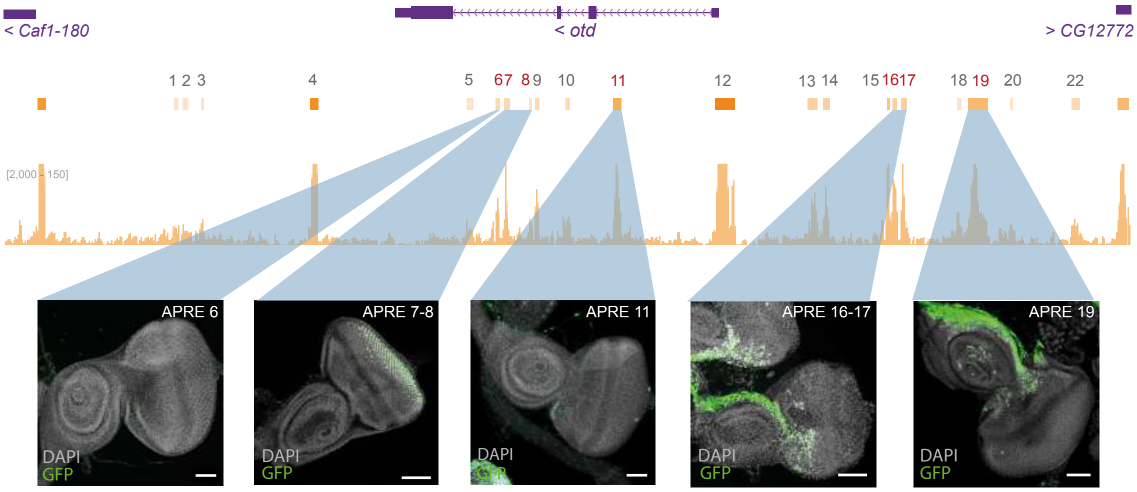

Figure S9. Activity of tested APREs in the *otd* locus of *D. simulans* and *D. mauritiana*. L3 instar eye-antenna imaginal discs from *D. melanogaster* lines containing *D. mauritiana-APRE4-GAL4, D. mauritiana-APRE6-GAL4, D. mauritiana-APRE7-8-GAL4, D. simulans-APRE11-GAL4, D. mauritiana-APRE13-14-GAL4, D. mauritiana-APRE16-17-GAL4, D. mauritiana-APRE19-GAL4* crossed to *UAS-GFP* and stained with DAPI (grey). Scale bar = 50um.
