# Supplementary material for: Heterochrony in orthodenticle expression is associated with ommatidial size variation between Drosophila species
Source: BMC Biol. 2025 Feb 4;23:34. doi: 10.1186/s12915-025-02136-8 (PMC11792340; doi:10.1186/s12915-025-02136-8)
Supplement: Supplementary file 18 — Additional file 18: Fig. S10. D. melanogaster, D. simulans and D. mauritiana otd-APRE7-8 enhancer activity in 3rd instar larvae eye imaginal discs. (a) D. melanogaster, D. simulans and D. mauritiana otd-APRE7-8 3rd instar larvae eye imaginal discs immunostained with anti-Elav antibody to visualise the progression of ommatidia maturation as proxy for the developmental time. (b) Plot showing the number of GFP-positive ommatidia rows (x-axis) activated by otd-APRE7-8 at different developmental time points (y-axis, developmental points inferred by number of ommatidia rows) for both species. (c) Violin plots showing the distance between MF and the first row of otd-APRE7-8 activity. D. mauritiana otd-APRE7-8 is active earlier during the differentiation of ommatidia. [file 12915_2025_2136_MOESM18_ESM.pdf]

**Figure S10**

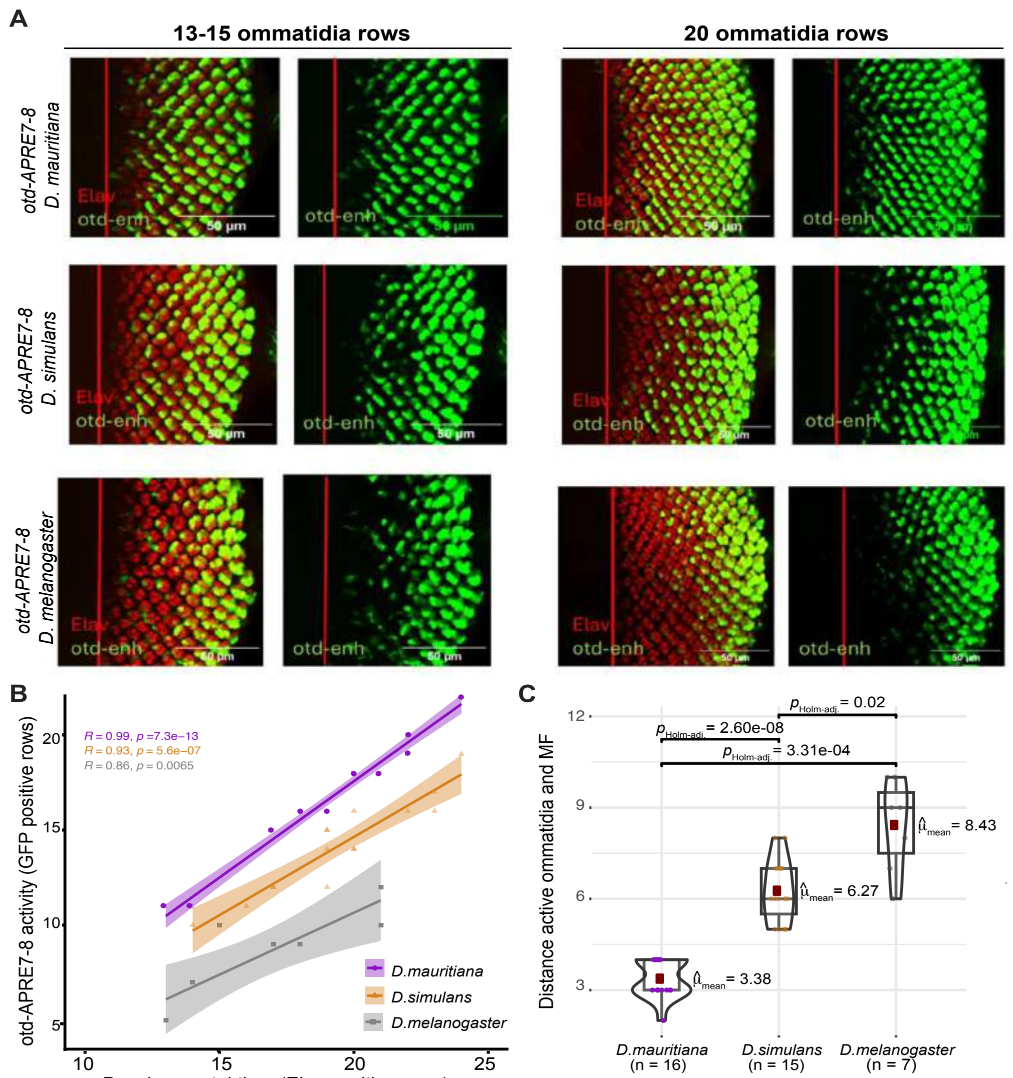


**Figure S10. *D. melanogaster, D. simulans* and *D. mauritiana otd-APRE7-8* enhancer activity in 3rd instar larvae eye imaginal discs. (A)** *D. melanogaster, D. simulans* and *D. mauritiana otd-APRE7-8* 3rd instar larvae eye imaginal discs immunostained with anti-Elav antibody to visualise the progression of ommatidia maturation as proxy for the developmental time. **(B)** Plot showing the number of GFP-positive ommatidia rows (x-axis) activated by *otd-APRE7-8* at different developmental time points (y-axis, developmental points inferred by number of ommatidia rows) for both species. **(C)** Violin plots showing the distance between MF and the first row of *otd-APRE7-8* activity. *D. mauritiana otd-APRE7-8* is active earlier during the differentiation of ommatidia.
