# Supplementary material for: Heterochrony in orthodenticle expression is associated with ommatidial size variation between Drosophila species
Source: BMC Biol. 2025 Feb 4;23:34. doi: 10.1186/s12915-025-02136-8 (PMC11792340; doi:10.1186/s12915-025-02136-8)
Supplement: Supplementary file 19 — Additional file 19: Dataset S1. Clustal Omega alignment of the 2.5 kb APRE7-8 region from selected strains of D. mauritiana, D. simulans and D. melanogaster including the sequences used in enhancer reporter constructs for each species (otd-APRE7-8). [file 12915_2025_2136_MOESM19_ESM.pdf]

```
                                                  Ct                So          Ct        Dve/Gl
D_melanogaster_OTD_enhancer        GACGTCCTCGTCTATTTGTTTAGGTGTCCACGATGATAGGGCCTAAATAAACAAATCCAA 60
Dsim_m3                            ---------CGTCTGTTTGTTTAGGTGTCCACGATGATAGGGCCTAAACAAACAGACCCAA 52
Dsim_w501                          -------AGCGTCTGTTTGTTTAGGTGTCCACGATGATAGGGCCTAAACAAACAGACCCAA 54
D_simulans_OTD_enhancer            --GACGTCCGTCTGTTTGTTTAGGTGTCCACGATGATAGGGCCTAAACAAACAGACCCAA 58
mau12                              -----TAGCGTCTGTTTGTTTAGGTGTCCACGATGATAGGGCCTAAACAAACAGACCCAA 55
Dmau_mav2                          -------GCGTCTGTTTGTTTAGGTGTCCACGATGATAGGGCCTAAACAAACAGACCCAA 53
D_mauritiana_OTD_enhancer          -GACGTCGCGTCTGTTTGTTTAGGTGTCCACGATGATAGGGCCTAAACAAACAGACCCAA 59
Dmau_tam16r                        -------GCGTCTGTTTGTTTAGGTGTCCACGATGATAGGGCCTAAACAAACAGACCCAA 53
Dmau_red3                          -------GCGTCTGTTTGTTTAGGTGTCCACGATGATAGGGCCTAAACAAACAGACCCAA 53
                                           ***** ********************************* ***** * ****

D_melanogaster_OTD_enhancer        TTCCCTAGCGCCGTTGGCCCGAAAAACTGCCAAATGGCCCAAAAACGATCACAGTTTCCG 120
Dsim_m3                            TTCCCTAGCGCCGTTGGCCCAAA-AACTGCCAAATGGCCCAAAAACGATCACAGTTTCCG 111
Dsim_w501                          TTCCCTAGCGCCGTTGGCCCAAA-AACTGCCAAATGGCCCAAAAACGATCACAGTTTCCG 113
D_simulans_OTD_enhancer            TTCCCTAGCGCCGTTGGCCCAAA-AACTGCCAAATGGCCCAAAAACGATCACAGTTTCCG 117
mau12                              TTCCCTAGCGCCGTTGGCCCAAA-AACTGCCAAATGGCCCAAAAACGATCACGGTTTCCG 114
Dmau_mav2                          TTCCCTAGCGCCGTTGGCCCAAA-AACTGCCAAATGGCCCAAAAACGATCACGGTTTCCG 112
D_mauritiana_OTD_enhancer          TTCCCTAGCGCCGTTGGCCCAAA-AACTGCCAAATGGCCCAAAAACGATCACGGTTTCCG 118
Dmau_tam16r                        TTCCCTAGCGCCGTTGGCCCAAA-AACTGCCAAATGGCCCAAAAACGATCACAGTTTCCG 112
Dmau_red3                          TTCCCTAGCGCCGTTGGCCCAAA-AACTGCCAAATGGCCCAAAAACGATCACAGTTTCCG 112
                                   ******************** ** **************************** *******
                                                        Hth                 Pph13
D_melanogaster_OTD_enhancer        GTCGTTGGAAAAAAAAAAAAAAAAACTGTCATCTCCTTTGGTAATTTCTTGGGCAAACAC 180
Dsim_m3                            GTCGCTGAAAAAACTG-----------TCATCTCCTTTTGGTAATTTCTTGGGCAAACAC 160
Dsim_w501                          GTCGCTGGAAAAACTG-----------TCATCTCCTTTTGGTAATTTCTTGGGCAAACAC 162
D_simulans_OTD_enhancer            GTCGCTGGAAAAACTG-----------TCATCTCCTTTTGGTAATTTCTTGGGCAAACAC 166
mau12                              GTCGCTGGAAAAACTG-----------TCATCTCCTTTTGGTAATTTCTTGGGCAAACAC 163
Dmau_mav2                          GTCGCTGGAAAAACTG-----------TCATCTCCTTTTGGTAATTTCTTGGGCAAACAC 161
D_mauritiana_OTD_enhancer          GTCGCTGGAAAAACTG-----------TCATCTCCTTTTGGTAATTTCTTGGGCAAACAC 167
Dmau_tam16r                        GTCGCTGGAAAAACTG-----------TCATCTCCTTTTGGTAATTTCTTGGGCAAACAC 161
Dmau_red3                          GTCGCTGGAAAAACTG-----------TCATCTCCTTTTGGTAATTTCTTGGGCAAACAC 161
                                   **** ** *****                  * ***********************
                                           Pnr                    Lz     Ci/Gl-
D_melanogaster_OTD_enhancer        AGCCGCAACATCGATCGTTTTTGGACACTGAAACTGCAATGAAAACACACAACGCCAGGC 240
Dsim_m3                            AGCCGCAACATCGATCGTTTTTGTGCACTGAAACTGCAATGAAAACACACAACGTCAGGC 220
Dsim_w501                          AGCCGCAACATCGATCGTTTTTGTACACTGCAACTGCAATGAAAACACACAACGTCAGGC 222
D_simulans_OTD_enhancer            AGCCGCAACATCGATCGTTTTTGTACACTGCAACTGCAATGAAAACACACAACGTCAGGC 226
mau12                              AGCCGCAACATCGATCGTTTTTGTACACTGCAACTGCAATGAAAACACACAACGCCAGGC 223
Dmau_mav2                          AGCCGCAACATCGATCGCTTTTGTACACTGCAACTGCAATGAAAACACACAACGTCAGGC 221
D_mauritiana_OTD_enhancer          AGCCGCAACATCGATCGTTTTTGTACACTGCAACTGCAATGAAAACACACAACGTCAGGC 227
Dmau_tam16r                        AGCCGCAACATCGATCGTTTTTGTACACTGCAACTGCAATGAAAACACACAACGTCAGGC 221
Dmau_red3                          AGCCGCAACATCGATCGTTTTTGTACACTGCAACTGCAATGAAAACACACAACGTCAGGC 221
                                   ***************** *****  ***** ************************ *****
                                                    Dve                       Ct      Pnr
D_melanogaster_OTD_enhancer        GAGTGTACAAAAAATAAACATTATTGGCAACAGAGCTACACATTGATCTATCGATTTGTT 300
Dsim_m3                            GAGCGCACAAAAAAATAAACATTATTGGCAACAGCTACAT----TGATCTATCGATTTGTT 276
Dsim_w501                          GAGCGCACAAAAAAATAAACATTATTGGCAACAGCTACAT----TGATCTATCGATTTGTT 278
D_simulans_OTD_enhancer            GAGCGCACAAAAAAATAAACATTATTGGCAACAGCTACAT----TGATCTATCGATTTGTT 282
mau12                              GAGCGCACAAAAAAATAAACATTATTGGCAACAGCTACAT----TGATCTATCGATTTGTT 279
Dmau_mav2                          GAGCGCACAAAAAAATAAACATTATTGGCAACAGCTACAT----TGATCTATCGATTTGTT 277
D_mauritiana_OTD_enhancer          GAGCGCACAAAAAAATAAACATTATTGGCAACAGCTACAT----TGATCTATCGATTTGTT 283
Dmau_tam16r                        GAGCGCACAAAAAAATAAACATTATTGGCAACAGCTACAT----TGATCTATCGATTTGTT 277
Dmau_red3                          GAGCGCACAAAAAAATAAACATTATTGGCAACAGCTACAT----TGATCTATCGATTTGTT 277
                                   *** * ***************************    *     *****************

D_melanogaster_OTD_enhancer        TGCTTTTCTTCTGCTTCTGCTTTTGCT----------------CTTGCTTTGCTTTTGCT 344
Dsim_m3                            TGCTTTTCTTCTGCTTCTGCTTTTGCTTTTGCCTTGCTT----------TTGCCTTGCTT 326
Dsim_w501                          TGCTTTTCTTCTGCTTCTGCTTTTACCTTGCTTTTGCTTTGCTTTTGCTTTGCTTTTGCT 338
D_simulans_OTD_enhancer            TGCTTTTCTTCTGCTTCTGCTTTTACCTTGCTTTTGCTTTGCTTTTGCTTTGCTTTTGCT 342
mau12                              TGCTTTTCTTCTGCTTCTGCTTCTGCTT-----TTGCTTTGCTTTTGCTTTGCTTTGCT- 333
Dmau_mav2                          TGCTTTTCTTCTGC------------TT-----TTGCTTTGCTTTTGCTTTGCTTTGCC- 319
D_mauritiana_OTD_enhancer          TGCTTTTCTTCTGCTTCTGCTTTTGCCTTGCTTTTGCTTTGCTTTTGCTTTGCTTTGCT- 342
Dmau_tam16r                        TGCTTTTCTTCTGCTTCTGCTTCTTCCTTGCTTTTGCTTTGCTTTTGCTTTGCTTTGCT- 336
Dmau_red3                          TGCTTTTCTTCTGCTTCTGCTTCTTCCTTGCTTTTGCTTTGCTTTTGCTTTGCTTTGCT- 336
                                   **************                                 **** **
   Sens/Ss low prob                                             Pph13
D_melanogaster_OTD_enhancer        TTGCTTTGCTTTGCTTGCCCTTGCTTTGTTAGCCGCTGCTTTTGTCCGTGTAATTTGAGG 404
Dsim_m3                            TTGCTTTGCTTTGCTTGCCCTTGCTTTGTTAGCCGCTGCTTTTGTCCGTGTAATTTGAGG 386
Dsim_w501                          TTGCTTTGCTTTGCTTGCCCTTGCTTTGTTAGCCGCTGCTTTTGTCCGTGTAATTTGAGG 398
D_simulans_OTD_enhancer            TTGCTTTGCTTTGCTTGCCCTTGCTTTGTTAGCCGCTGCTTTTGTCCGTGTAATTTGAGG 402
mau12                              ----TTTGCTTTGCTTGGCCTTGCTTTGTTAGCCGCTGCTTTTGTCCGTGTAATTTGAGG 389
Dmau_mav2                          ----TTTGCTTTGCTTGGCCTTGCTTTGTTAGCCGCTGCTTTTGTCCGTGTAATTTGAGG 375
D_mauritiana_OTD_enhancer          ----TTTGCTTTGCTTGCCCTTGCTTTGTTAGCCGCTGCTTTTGTCCGTGTAATTTGAGG 398
Dmau_tam16r                        ----TTTGCTTTGCTTGCCCTTGCTTTGTTAGCCGCTGCTTTTGTCCGTGTAATTTGAGG 392
Dmau_red3                          ----TTTGCTTTGCTTGGCCTTGCTTTGTTAGCCGCTGCTTTTGTCCGTGTAATTTGAGG 392
                                       ************* ***************************************
```

```
                                 1

                                     Hth        Otd        Gl                      Otd
D_melanogaster_OTD_enhancer      ACGTGACACTGCAGGGTTAGAATCATGCAACTTTCATCAGGCTTATGAAAGATATAAACA 464
Dsim_m3                          ACGTGACACTGCAGGGCTAGAATCATGCAACTTTCATCGGACTAAAGAAGGATATAAACA 446
Dsim_w501                        ACGTGACACTGCAGGGCTAGAATCATGCAACTTTCATCGGACTAAAGAAGGATATAAACA 458
D_simulans_OTD_enhancer          ACGTGACACTGCAGGGCTAGAATCATGCAACTTTCATCGGACTAAAGAAGGATATAAACA 462
mau12                            ACGTGACACTGCAGGGCTAGAATCATGCAACTTTCATCAGGCTTAAGAAAGATATAAACC 449
Dmau_mav2                        ACGTGACACTGCAGGGCTAGAATCATGCAACTTTCATCAGGCTTAAGAAAGATATAAACC 435
D_mauritiana_OTD_enhancer        ACGTGACACTGCAGGGCTAGAATCACGCAACTTTCATCAGGCTTAAGAAAGATATAAACA 458
Dmau_tam16r                      ACGCGACACTGCAGGTCTAGAATCATGCAACTTTCATCAGGCTTAAGAAAGATATAAACA 452
Dmau_red3                        ACGCGACACTGCAGGGCTAGAATCATGCAACTTTCATCAGGCTTAAGAAAGATATAAACA 452
                                 *** ***********  ******** ************ * ** * *** *********

                                        Luna/So/Lz                    So
D_melanogaster_OTD_enhancer      GAAACTTCTGTAACAAGACAACTAACTTTAGTATAAGTTGATAAGTTAGTAGAGCGCTCT 524
Dsim_m3                          GGAGTTTCTGCAACAAGACAACTAACTTTAGAATATCAGTATAAGTTGATAAGTTGAAGA 506
Dsim_w501                        GGAGTTTCTGCAACAAGACAACTAACTTTAGAATATCAGTATAAGTTGATAAGTTGAAGA 518
D_simulans_OTD_enhancer          GGAGTTTCTGCAACAAGACAACTAACTTTAGAATATCAGTATAAGTTGATAAGTTGAAGA 522
mau12                            GGAGTTTCTCCAACAAGACGGCTAACTTTAGAATATCAGTATAAGTTGATAAGTTGAAGA 509
Dmau_mav2                        GGAGTTTCTCCAACAAGACGGCTAACTTTAGAATATCAGTATAAGTTGATAAGTTGAAGA 495
D_mauritiana_OTD_enhancer        GGAGTTTCTCCAACAAGACGGCTAACTTTAGAATATCAGTATAAGTTGATAAGTTGAAGA 518
Dmau_tam16r                      GGAGTTTCTCCAACAAGACGGCTAACTTTAGAATATCAGTATAAGTTGATAAGTTGAAGA 512
Dmau_red3                        GGAGTTGCTCTAACAAGACGGCTAACTTTGGAATATCAGTATAAGTTGATAAGTTGAAGA 512
                                 * *  * **  ********  ******** * ***      *******  **    *
                                              2

                                                                                  So
D_melanogaster_OTD_enhancer      AAAGATAAAGATAAAAACAAGAGATAAAAGATATGAGAGTAAGGATACCAAGTTA--GCT 582
Dsim_m3                          TATGAGCGT-------------------------------AAGGATACCGAGTTAGCGCT 535
Dsim_w501                        TATGAGCGT-------------------------------AAGGATACCGAGTTAGCGCT 547
D_simulans_OTD_enhancer          TATGAGCGT-------------------------------AAGGATACCGAGTTAGCGCT 551
mau12                            TATGAGAGT-------------------------------AAGGATACCGAGTTA--GCT 536
Dmau_mav2                        TATGAGAGT-------------------------------AAGGATACCGAGTTA--GCT 522
D_mauritiana_OTD_enhancer        TATGAGAGT-------------------------------AAGGATACCGAGCTA--GCT 545
Dmau_tam16r                      TATGAGAGT-------------------------------AAGGATACCGAGTTA--GCT 539
Dmau_red3                        TATGAGAGT-------------------------------AAGGATATCGAGTTA--GCT 539
                                  * **                                  ******* * ** **  ***

D_melanogaster_OTD_enhancer      ACTTAGATAACGAGCACGCT---------TTAAAAGAAAGAAAGAAAGAAAGGCAGTTAA 633
Dsim_m3                          AGTTAGATAACCAGCAGGCTTTAAAAGACAGTCGAATAACAGTGAAAGAAAGGCAGTTAA 595
Dsim_w501                        AGTTAGATAACCAGCAGGCTTTAAAAGGCAGTCGAATAACAGTGAAAGAAAGTCAGTTAA 607
D_simulans_OTD_enhancer          AGTTAGATAACCAGCAGGCTTTAAAAGGCAGTCGAATAACAGTGAAAGAAAGTCAGTTAA 611
mau12                            AGTTAGATAACCAGCAGGCTTTAAAAGACAGTCGCATAACAGTGAAAGAAAGTCAGTTAA 596
Dmau_mav2                        AGTTAGATAACCAGCAGGCTTTAAAAGACAGTCGCATAACAGTGAACGAAAGTCAGTTAA 582
D_mauritiana_OTD_enhancer        AGTTAGATAACCAGCAGGCTTTAAAAGACAGTCGCATAACAGTGAAAGAAAGTCAGTTAA 605
Dmau_tam16r                      AGTTAGATAACCAGCAGGCTTTAAAAGACAGTCACATAACAGTGAAAGAAAGTCAGTTAA 599
Dmau_red3                        AGTTAGATAACCAGCAGGCTTTAAAAGAA-------TAACAGTGAAAGAAAGTCAGTTAA 592
                                 * ********* **** ***                ** *  *** ***** *******

                                                                      Otd      Gl
D_melanogaster_OTD_enhancer      TAGCAAGTCAACTTTAAAGACCTGGCCGTAAAAAGCAACAGCGATTAAGGAGGAAGCCCC 693
Dsim_m3                          TAGCAAGTCAACTTTAAAGACCTGGCCGGAAAAAAAAGCAACAGCGATTAAGGAAGCCCC 655
Dsim_w501                        TAGCAAGTCAACTTTAAAGACCTGGCCGGAAAAAAAAGCAACAGCGATTAAGGAAGCCCC 667
D_simulans_OTD_enhancer          TAGCAAGTCAACTTTAAAGACCTGGCCGGAAAAAAAAGCAACAGCGATTAAGGAAGCCCC 671
mau12                            TAGCAAGTCAACTTTAAAGACCTGGCCGGAAAAAAAAGCAACAGCGATTAAGGAAGCCCC 656
Dmau_mav2                        TAGCAAGTCAACTTTAAAGACCTGGCCGGAAA-AAAAGCAACAGCGATTAAGGAAGCCCC 641
D_mauritiana_OTD_enhancer        TAGCAAGTCAACTTTAAAGACCTGGCCGGAAA-AAAAGCAACAGCGATTAAGGAAGCCCC 664
Dmau_tam16r                      AAGCAAGTCAACTTTAAAGACCTGGCCGGAAA-AAAAGCAACAGCGATTAAGGAAGCCCC 658
Dmau_red3                        AAGCAAGTCAACTTTAAAGACCTGGCCGGAAA-AAAAGCAACAGCGATTAAGGAAGCCCC 651
                                  *************************** *** *   * ** *     *   **********

                                                                                   Luna
D_melanogaster_OTD_enhancer      TGCAGTGGTCGGCTCCGAATGAAAAACGACACGTTCTATTTTCGAAGGGGTGGGGAGGGG 753
Dsim_m3                          TGCAGTGGTCGGCTCCGAATGAAAAACGACACGTTCTATTTTCGAAGGGGAGGGGCA--- 712
Dsim_w501                        TGCAGTGGTCGGCTCCGAATGAAAAACGACACGTTCTATTTTCGTAGGGGAGGGGCA--- 724
D_simulans_OTD_enhancer          TGCAGTGGTCGGCTCCGAATGAAAAACGACACGTTCTATTTTCGTAGGGGAGGGGCA--- 728
mau12                            TGCAGTGGTCGGCTCCGAATGAAAAACGACACGTTCTATTTTCGAAGGGGAGGGGCA--- 713
Dmau_mav2                        TGCAGTGGTCGGCTCCGAATGAAAAACGACACGTTCTATTTTCGAGG-GGAGGGGCA--- 697
D_mauritiana_OTD_enhancer        TGCAGTGGTCGGCTCCGAATGAAAAACGACACGTTCTATTCTCGAAGGGGAGGGGCA--- 721
Dmau_tam16r                      TGCAGTGGTCGGCTCCGAATGAAAAACGACACGTTCTATTTTCGAAGGGGAGGGGCA--- 715
Dmau_red3                        TGCAGTGGTCGGCTCCGAATGAAAAACGACACGTTCTATTTTCGAAGGGGAGGGGCA--- 708
                                 **************************************** ***  * ** ****

                                                                                     Ato
D_melanogaster_OTD_enhancer      GAATGCAGACCGAATGAATGGTGAGCATGGAAAGAATACAGCAAGGACACCTTGCACATG 813
Dsim_m3                          -------GCGGGAATGAATGGTGAGCAGGGAAAGAATACGGCAAGGACACCTTGCACATG 765
Dsim_w501                        -------GCGGGAATGAATGGTGAGCAGGGAAAGAATACAGCAAGGACACCTTGCACATG 777
D_simulans_OTD_enhancer          -------GCGGGAATGAATGGTGAGCAGGGAAAGAATACAGCAAGGACACCTTGCACATG 781
mau12                            -------GCGGGAATGAATGGTGAGCAGGGAAAGAATACAGCAAGGACACCTTGCACATG 766
Dmau_mav2                        -------GCGGGAATGAATGGTGAGCAGGGAAAGAATACAGCAAGGACACCTTGCACATG 750
```

```
D_mauritiana_OTD_enhancer     -------GCGGGAATGAATGGTGAGCAGGGAAAGAATACAGCAAGGACACCTTGCACATG 774
Dmau_tam16r                   -------GCGGGAATGAATGGTGAGCAGGGAAAGAATACAGCAAGGACACCTTGCACATG 768
Dmau_red3                     -------GCGGGAATGAATGGTGAGCAGGGAAAGAATACAGCAAGGACACCTTGCACATG 761
                                     *   **************** *********** ********************

D_melanogaster_OTD_enhancer   TCCCATGAATCGC-CTGCTACTGTATTGTTTCCTTTTCAAAGTTACGTTTTTTACACAAT 872
Dsim_m3                       TCCCATGAATCGGCTGGCTACTGTATTGTTTCCTTTTCAAAGTTACGTTTTTTACACAAT 825
Dsim_w501                     TCCCATGAATCGGCTGGCTACTGTATTGTTTCCTTTTCAAAGTTACGTTTTTTACACAAT 837
D_simulans_OTD_enhancer       TCCCATGAATCGGCTGGCTACTGTATTGTTTCCTTTTCAAAGTTACGTTTTTTACACAAT 841
mau12                         TCCCATGAATCGGCCGGCTACTGTATTGTTTCCTTTTCAAAGTTACGTTTTTTACACAAT 826
Dmau_mav2                     TCCCATGAATCGGCCGGCTACTGTATTGTTTCCTTTTCAAAGTTACGTTTTTTACACAAT 810
D_mauritiana_OTD_enhancer     TCCCATGAATCGGCTGGCTACTGTATTGTTTCCTTTTCAAAGTTACGTTTTTTACACAAT 834
Dmau_tam16r                   TCCCATGAATCGGCTGGCCACTGTATTGTTTCCTTTTCAAAGTTACGTTTTTTACACAAT 828
Dmau_red3                     TCCCATGAATCGGCTGGCCACTGTATTGTTTCCTTTTCAAAGTTACGTTTTTTACACAAT 821
                              ************    ** *****************************************

                                            Ey
D_melanogaster_OTD_enhancer   TGATTCGAGTAATGCACACCGGCGAAACCAAGGGGCGATATACATATGTATGTGTATAAG 932
Dsim_m3                       TGATTCCAGTAATGCACACCGGAGAAACCAGGGGGCAAGATATATGTATGTATATGT--- 882
Dsim_w501                     TGATTCCAGTAATGCACACCGGAGAAACCAGGGGGCAAGATATATGTATGTGTATGT--- 894
D_simulans_OTD_enhancer       TGATTCCAGTAATGCACACCGGAGAAACCAGGGGGCAAGATATATGTATGTATATGT--- 898
mau12                         TGATTCCAGTAATGCACACCGGAGAAACCAGGGGGCAAGATATATGTATGTGTAAA---- 882
Dmau_mav2                     TGATTCCAGTAATGCACACCGGAGAAACCAGGGGGCAAGATATATGTGTGTGTAAA---- 866
D_mauritiana_OTD_enhancer     TGATTCCAGTAATGCACACCGGAGAAACCAGGGGGCAAGATATATGTGTGTGTAAA---- 890
Dmau_tam16r                   TGATTCCAGTAATGCACACCGGAGAAACCAGGGGGCAAGATATATGTGTGTGGAAA---- 884
Dmau_red3                     TGATTCCAGTAATGCACACCGGAGAAACCAGGGGGCAAGATATATGTGTGTGTAAA---- 877
                              ****** *************** ******* ***** * *** ** * * *

                                                                     Luna        Svp? Hth
D_melanogaster_OTD_enhancer   GAAGTGTGGATGGATGTACTATATATGAATGTCGGGGGTGTAGCTGGGAATTGACATTTG 992
Dsim_m3                       -----------GTAAAGGAAGTATGTGGATGTCGGGGGTGTAGCTGGGAATTGACATTTG 931
Dsim_w501                     -----------GTAAAGGAAGTATGTGGATGTCGGGGGTGTAGCTGGGAATTGACATTTG 943
D_simulans_OTD_enhancer       -----------GTAAAGGAAGTATGTGGATGTCGGGGGTGTAGCTGGGAATTGACATTTG 947
mau12                         --------------GGAAGTGTGGATGGATGTCGGGGGTGTAGCTGGGAATTGGCATTTG 928
Dmau_mav2                     --------------GGAAGTGTGGATGGATGTCGGGGGTGTAGCTGGGAATTGGCATTTG 912
D_mauritiana_OTD_enhancer     --------------GGAAGTGTGGATGGATGTCGGGGGTGTAGCTGGGAATTGGCATTTG 936
Dmau_tam16r                   --------------GGAAGTGTGGATGGATGTCGGGGGTGTAGCTGGGAATTGGCATTTG 930
Dmau_red3                     --------------GGAAGTGTGGATGGATGTCGGGGGTGTAGCTGGGAATTGGCATTTG 923
                                                   *   ** ************************* ******

                                            3                                      4

                              Sens?/Pnr               Ato
D_melanogaster_OTD_enhancer   TATAGATTTTCACATTTTTCCACACATGTCGCTTCTTCTTGGTTAGTTTCTTGCGTTGTT 1052
Dsim_m3                       TATAGATTTCCCTGTT--TCCACACATGTCGCTTCTTCTTGGATAGTTCGCCCTGGCTCG 989
Dsim_w501                     TATAGATTTCCCTGTT--TCCACACATGTCGCTTCTTCTTGGATAGTTCGCCCTGGCTTG 1001
D_simulans_OTD_enhancer       TATAGATTTCCCTGTT--TCCACACATGTCGCTTCTTCTTGGATAGTTCGCCCTGGCTTG 1005
mau12                         TATAGATTTCCCTGTT--TCCACACATGTCGCTTCTTCTTGGATAGTTCGCCCTGGCTTG 986
Dmau_mav2                     TATAGATTTCCCTGTT--TCCACACATGTCGCTTCTTCTTGGATAGTTCGCCCTGGCTTG 970
D_mauritiana_OTD_enhancer     TATAGATTTCCCTGTT--TCCACACATGTCGCTTCTTCTTGGATAGTTCGCCCTGGCTTG 994
Dmau_tam16r                   TATAGATTTCCCTGTT--TCCACACATGTCGCTTCTTCTTGGATAGTTCGCCCTGGCTTG 988
Dmau_red3                     TATAGATTTCCCTGTT--TCCA--CATGTCGCTTCTTCTTGGATAGTTCGCCCTGGCTTG 979
                              ********* *   **  ****  ****************** *****      *

                                            Sens?/Ro
D_melanogaster_OTD_enhancer   CTTGTCGCCACGGCTTGATTGATTTTCAAATGCGAGTACAGTGGTTGCATCATCGGAATA 1112
Dsim_m3                       ACTGATTTTC--AAATGCTTTTCAGACGCAGTCGAGCACAGTGGTTGCATCATAGGGAAA 1047
Dsim_w501                     ACTGATTCTC--AAATGCTTTTCAGACGCAGTCGAGCACAGTGGTTGCATCATAGGGAAA 1059
D_simulans_OTD_enhancer       ACTGATTCTC--AAATGCTTTTCAGACGCAGTCGAGCACAGTGGTTGCATCATAGGGAAA 1063
mau12                         ACTGATTCTC--AAATGCTTTTCAGACGCAGCCGAGCACAGTGGTTGCATCATGGGGAAA 1044
Dmau_mav2                     ACTGATTCTC--AAATGCTTTTCAGACGCAGCCGAGCACAGTGGTTGCATCATGGGGAAA 1028
D_mauritiana_OTD_enhancer     ACCGATTCTC--AAATGCTTTTCAGACGCAGCCGAGCACAGTGGTTGCATCATGGGGAAA 1052
Dmau_tam16r                   ACCGATTCTC--AAATGCTTTTCAGACGCAGCCGAGCACAGTGGTTGCATCATGGGGAAA 1046
Dmau_red3                     ACTGATTCTC--AAATGCTTTTCAGACGCAGCCGAGCACAGTGGTTGCATCATGGGGAAA 1037
                                 *           ** **      *  *  **** **************** ** * *

                                                                         5

                                            So/Gl-                Pph13/En/Oc/Dve?
D_melanogaster_OTD_enhancer   TTTCAA------------------------TGATATTTACTAAATCGTGTGCAAATTTCT 1148
Dsim_m3                       AGCAAAGATATTTACTAAAAAATATCGTATTAATTCGTGTTAATTCGTTTGCAAATATCA 1107
Dsim_w501                     AGCAAAGATATTTACTAAAAAATATCGTATTAATTCGTGTTAATTCGTTTGCAAATATCA 1119
D_simulans_OTD_enhancer       AGCAAAGATATTTACTAAAAAATATCGTATTAATTCGTGTTAATTCGTTTGCAAATATCA 1123
mau12                         AGCAAAGATATTAACTAAAAAATTTCGTATTAATTGTGTTAATT-CGTTTGCAAATATAT 1103
Dmau_mav2                     AGCAAAGATATTAACTAAAAAATTTCGTATTAATTGTGTTAATT-CGTTTGCAAATATAT 1087
D_mauritiana_OTD_enhancer     AGCAAAGATATTAACTAAAAAATGTCGTATTAATTGTGTTAATT-CGTTTGCAAATATAT 1111
Dmau_tam16r                   ATCAAAGATATTAACTAAAAAATATCGTATTAATTGTGTTAATT-CGTTTGCAAATATAT 1105
Dmau_red3                     ATCAAAGATATTAACTAAAAAATATCGTATTAATTGTGTTAATT-CGTTTGCAAATATAT 1096
                                  **                        * **       *   *** ******* *
                                                             6
```

```
                                                  So/So       Pnr/Otd       Sens?/Otd
D_melanogaster_OTD_enhancer     TGCAA------ATATCATATGATATAGTACCGATTACATTCAAATCTTAACCCCATTTCG 1202
Dsim_m3                         TAT--------AATATAATTCGGATAAAGTACCTGAAGTACCTATCTTAGCCCCATTTGG 1159
Dsim_w501                       TAT--------AATATAATTCGGATAAAGTACCTGAAGTACCTATCTTAGCCCCATTTGG 1171
D_simulans_OTD_enhancer         TAT--------AATATAATTCGGATAAAGTACCTGAAGTACCTATCTTAACCCCATTTGG 1175
mau12                           AAGATCATACAATTCTCATTATGATATGTACCTATGGATTCATATCTTAGCCCCATTTGG 1163
Dmau_mav2                       AAGATCATACAATTCTCATTATGATATGTACCTATGGATTCATATCTTAGCCCCATTTGG 1147
D_mauritiana_OTD_enhancer       AAGATCATACAATTCTCATTA-----TGTACCTAAGGATTCATATCTTAGCCCCATTTGG 1166
Dmau_tam16r                     AAGATCATACAATTCTCATTATGATATGTACCTGAGGATTCATATCTTAGCCCAATTTGG 1165
Dmau_red3                       AAGATCATACAATTCTCATTA-----TGTACCTATGGATTCATATCTTAGCCCAATTTGG 1151
                                           *       *                  * *  ****** *** **** *
                                                     7
                                                           Sens? Hth
D_melanogaster_OTD_enhancer     TGCCACTGTACCT----CTGGGTAGTAACGCAACATGTCAATGCATCCATCCACTGTTAT 1258
Dsim_m3                         AGCCACTGTACCTGTGCCTCTGTAGCAACGCAACATGTCAATGCATCCATCCACTCTTAT 1219
Dsim_w501                       AGCCACTGTACCTGTGCCTCTGTAGCCACGCAACATGTCAATGCATCCATCCACTCTTAT 1231
D_simulans_OTD_enhancer         AGCCACTGTACCTGTGCCTCTGTAGCCACGCAACATGTCAATGCATCCATCCACTCTTAT 1235
mau12                           AGCCACTGTACCTGTGCCTCCGTAGCCACGCAACATGTCAATGCATCCATCCACTCTTAT 1223
Dmau_mav2                       AGCCACTGTACCTGTGCCTCTGTAGCCACGCAACATGTCAATGCATCCATCCACTCTTAT 1207
D_mauritiana_OTD_enhancer       AGCCACTGTACCTGTGCCTCTGTAGCCACGCAACATGTCAATGCATCCATCCACTCTTAT 1226
Dmau_tam16r                     AGCCACTGTACCTGTGCCTCTGTAGCCACGCAACATGTCAATGCATCCATCCACTCTTAT 1225
Dmau_red3                       AGCCACTGTACCTGTGCCTCTGTAGCCACGCAACATGTCAATGCATCCATCCACTCTTAT 1211
                                 ************    **  ****  **************************** ****
                                                Ci                 So/Dve/Otd
D_melanogaster_OTD_enhancer     AAAAACACATAGGCACCCACA----CTCGGTCACGTATCAGATTACAGGCAGGAAAACTA 1314
Dsim_m3                         AAAAACACATAGCCACCCACACTCGGTCGGTCACGTATCAGATTACAGGCAGGAAAACTA 1279
Dsim_w501                       AAAAACACATAGCCACCCACACTCGGTCGGTCACGTATCAGATTACAGGCAGGAAAACTA 1291
D_simulans_OTD_enhancer         AAAAACACATAGCCACCCACACTCGGTCGGTCACGTATCAGATTACAGGCAGGAAAACTA 1295
mau12                           AAAAACACATAGCCACCCACAGTCGGGCGGTCACGTATCAGATTACAGGCAGGAAAACTA 1283
Dmau_mav2                       AAAAACACATAGCCACCCACACTCGGGCGGTCACGTATCAGATTACAGGCAGGAAAACTA 1267
D_mauritiana_OTD_enhancer       AAAAACACATAGCCACCCACACTCGGTCGGTCACGTATCAGATTACAGGCAGGAAAACTA 1286
Dmau_tam16r                     AAAAACACATAGCCACCCACACTCGGGCGGTCACGTATCAGATTACAGGCAGGAAAACTA 1285
Dmau_red3                       AAAAACACATAGCCACCCACACTCGGGCGGTCACGTATCAGATTACAGGCAGGAAAACTA 1271
                                ************ ********      *********************************
                                              Lz
D_melanogaster_OTD_enhancer     GGAAAAACCTTAAAAAAACCCCAACCGACTGAAAACTGCGAATGGAAATGGAAATGCTGGC 1374
Dsim_m3                         GGAAAAACCTTACAAAACCCTAACCGACTGAAAACTGCGAATGGAAATGCTGGC------ 1333
Dsim_w501                       GGAAAAACCTTACAAAACCCCAACCGACTGAAAACTGCGAATGGAAATGCTGGC------ 1345
D_simulans_OTD_enhancer         GGAAAAACCTTACAAAACCCCAACCGACTGAAAACTGCGAATGGAAATGCTGGC------ 1349
mau12                           GGAAAAACCTTACAAAACCCCAACCGACTGAAAACTGCGAATGGAAATGCTGGC------ 1337
Dmau_mav2                       GGAAAAACCTTACAAAACCCCAACCGACTGAAAACTGCGAATGGAAATGCTGGC------ 1321
D_mauritiana_OTD_enhancer       GGAAAAACCTTACAAAACCCCAACCGACTGAAAACTGCGAATGGAAATGCTGGC------ 1340
Dmau_tam16r                     GGAAAAACCTTACAAAACCCCAACCGACTGAAAACTGCGAATGGAAATGCTGGC------ 1339
Dmau_red3                       GGAAAAACCTTACAAAACCCCAACCGACTGAAAACTGCGAATGGAAATGCTGGC------ 1325
                                ************ ******* ****************************

                                                                   Sens low prob
D_melanogaster_OTD_enhancer     CAAAAAGGCGGGGAAAGAGTAGCAGCAGTTGGGGCAAAAAACACGATTCGCTTGCATGTT 1434
Dsim_m3                         -CAAAAGGCGGGGCAAGAGGAGCAGCAGCT-GGGGCAAAAACACGATTCGTTTGCATGTT 1391
Dsim_w501                       -CAAAAGGCGGGGCAAGAGGAGCAGCAGCT-GGGGCAAAAACACGATTCGTTTGCATGTT 1403
D_simulans_OTD_enhancer         -CAAAAGGCGGGGCAAGAGGAGCAGCAGCT-GGGGCAAAAACACGATTCGTTTGCATGTT 1407
mau12                           -CAAAAGGCGGGGCAAGAGGAGCAGCAGCT-GGGGCAAAAACACGATTCGTTTGCATGTT 1395
Dmau_mav2                       -CAAAAGGCGGGGCAAGAGGAGCAGCAGCT-GGGGCAAAAACACGATTCGTTTGCATGTT 1379
D_mauritiana_OTD_enhancer       -CAAAAGGCGGGGCAAGAGGAGCAGCAGCT-GGGGCAAAAACACGATTCGTTTGCATGTT 1398
Dmau_tam16r                     -CAAAAGGCGGGGCAAGAGGAGCAGCAGCT-GGGGCAAAAACACGATTCGTTTGCATGTT 1397
Dmau_red3                       -CAAAAGGCGGGGCAAGAGGAGCAGCAGCT-GGGGCAAAAACACGATTCGTTTGCATGTT 1383
                                  *********** ***** ******** * ***  ************** *********

D_melanogaster_OTD_enhancer     ACAAGGCGTCTGGCTTTTGGTTTTCGTTTTGGCTTGGCTTTCGGTTTGGGTAACTACTGG 1494
Dsim_m3                         ACAAGGCGTCTGGCTTTTGGTTTTCGTTTTGGCTTGGCTTTCGGTTTGGGGAACCACTGG 1451
Dsim_w501                       ACAAGGCGTCTGGCTTTTGGTTTTCGTTTTGGCTTGGCTTTCGGTTTGGGGAACCACTGG 1463
D_simulans_OTD_enhancer         ACAAGGCGTCTGGCTTTTGGTTTTCGTTTTGGCTTGGCTTTCGGTTTGGGGAACCACTGG 1467
mau12                           ACAAGGCGTCTGGCTTTTGGTTTTCGTTTTGGCTTGGCTTTCGGTTTGGGGAACCACTGG 1455
Dmau_mav2                       ACAAGGCGTCTGGCTTTTGGTTTTCGTTTTGGCTTGGCTTTCGGTTTGGGGAACCACTGG 1439
D_mauritiana_OTD_enhancer       ACAAGGCGTCTGGCTTTTGGTTTTCGTTTTGGCTTGGCTTTCGGTTTGGGGAACCACTGG 1458
Dmau_tam16r                     ACAAGGCGTCTGGCTTTTGGTTTTCGTTTTGGCTTGGCTTTCGGTTTGGGGAACCACTGG 1457
Dmau_red3                       ACAAGGCGTCTGGCTTTTGGTTTTCGTTTTGGCTTGGCTTTCGGTTTGGGGAACCACTGG 1443
                                ************************************************** *** *****

D_melanogaster_OTD_enhancer     GTATTCAGCATTGGGGATCC 1514
Dsim_m3                         GTATTCAGCATTGGGGATCC 1471
```

```
Dsim_w501                       GTATTCAGCATTGGGGATCC 1483
D_simulans_OTD_enhancer         GTATTCAGCATTGGGGATCC 1487
mau12                           GTATTCAGCATTGGGGATCC 1475
Dmau_mav2                       GTATTCAGCATTGGGGATCC 1459
D_mauritiana_OTD_enhancer       GTATTCAGCATTGGGGATCC 1478
Dmau_tam16r                     GTATTCAGCATTGGGGATCC 1477
Dmau_red3                       GTATTCAGCATTGGGGATCC 1463
                                ********************
```
