# Supplementary material for: Heterochrony in orthodenticle expression is associated with ommatidial size variation between Drosophila species
Source: BMC Biol. 2025 Feb 4;23:34. doi: 10.1186/s12915-025-02136-8 (PMC11792340; doi:10.1186/s12915-025-02136-8)
Supplement: Supplementary file 20 — Additional file 20: Fig. S11. Schematic showing the position of the 2.5 kb APRE7-8 within the otd locus on the X chromosome of D. melanogaster. A 1.5 kb region shown to drive similar expression to the 2.5 kb region is indicated by a purple bar (37). Below is shown the ATAC-seq profile for this region and the positions of potential fixed mutations specific to D. mauritiana (arrows indicate SNPs and rectangles indicate short indels) with predicted binding sites for Otd, So and Cut in D. melanogaster indicated by arrows. Binding sites indicated with an asterisk contain mutations in D. mauritiana. [file 12915_2025_2136_MOESM20_ESM.pdf]

Figure S11

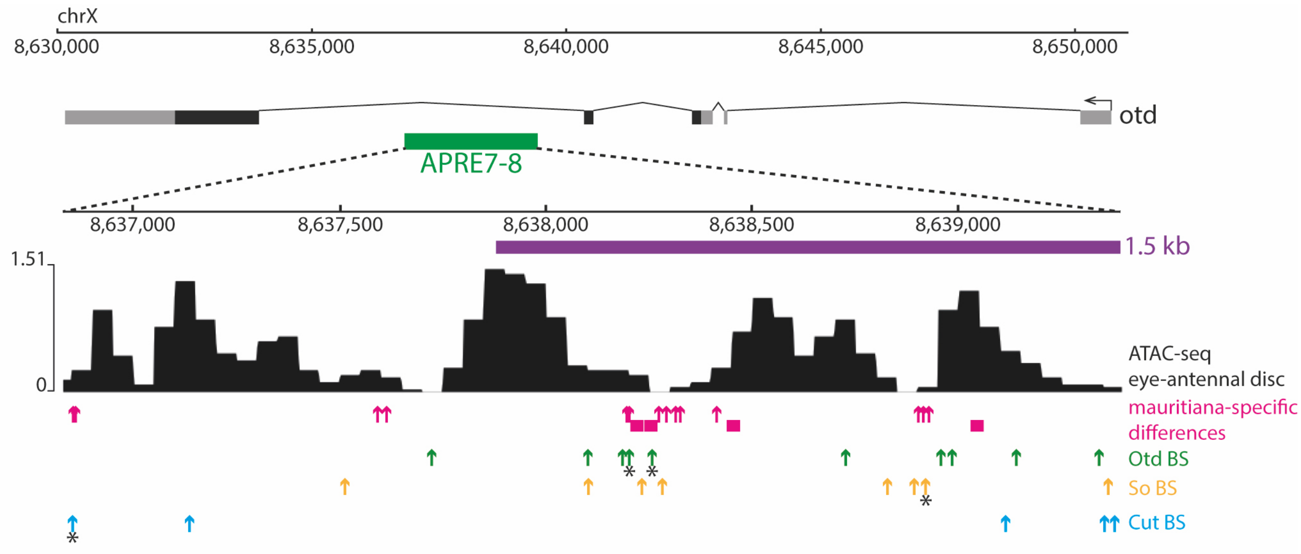

Figure S11. Schematic showing the position of the 2.5 kb APRE7-8 within the *otd* locus on the X chromosome of *D. melanogaster*. A 1.5 kb region shown to drive similar expression to the 2.6 kb region is indicated by a purple bar (37). Below is shown the ATAC-seq profile for this region and the positions of potential fixed mutations specific to *D. mauritiana* (arrows indicate SNPs and rectangles indicate short indels) with predicted binding sites for Otd, So and Cut in *D. melanogaster* indicated by arrows. Binding sites indicated with an asterisk contain mutations in *D. mauritiana*.
